# DNA methylation of blood cells is associated with prevalent type 2 diabetes in a meta-analysis of four European cohorts

**DOI:** 10.1186/s13148-021-01027-3

**Published:** 2021-02-23

**Authors:** Diana L. Juvinao-Quintero, Riccardo E. Marioni, Carolina Ochoa-Rosales, Tom C. Russ, Ian J. Deary, Joyce B. J. van Meurs, Trudy Voortman, Marie-France Hivert, Gemma C. Sharp, Caroline L. Relton, Hannah R. Elliott

**Affiliations:** 1 MRC Integrative Epidemiology, Bristol Medical School, Bristol, BS8 2BN UK; 2 Population Health Sciences, Bristol Medical School, University of Bristol, Bristol, BS8 2BN UK; 3 Centre for Genomic and Experimental Medicine, Institute of Genetics and Molecular Medicine, University of Edinburgh, Edinburgh, EH4 2XU UK; 4 Department of Epidemiology, Erasmus MC University Medical Center, Rotterdam, 3000 CA The Netherlands; 5 Centro de Vida Saludable de La Universidad de Concepción, Victoria 580, Concepción, Chile; 6 Alzheimer Scotland Dementia Research Centre, University of Edinburgh, 7 George Square, Edinburgh, EH8 9JZ UK; 7 Edinburgh Dementia Prevention Research Group, University of Edinburgh, Edinburgh, EH16 4UX UK; 8 Lothian Birth Cohorts, University of Edinburgh, Edinburgh, EH8 9JZ UK; 9 Department of Psychology, University of Edinburgh, Edinburgh, EH8 9JZ UK; 10 Department of Internal Medicine, Erasmus MC University Medical Center, Rotterdam, 3000 CA The Netherlands; 11 Division of Chronic Disease Research Across the Lifecourse, Department of Population Medicine, Harvard Medical School and Harvard Pilgrim Health Care, Boston, MA 02215 USA; 12 Bristol NIHR Biomedical Research Centre, Oakfield House, Oakfield Grove, Bristol, BS8 2BN UK; 13 MRC Integrative Epidemiology Unit, Population Health Sciences, Bristol Medical School, University of Bristol, Oakfield House, Oakfield Grove, Bristol, BS8 2BN UK

**Keywords:** DNA methylation, Prevalent T2D, Meta-analysis, ALSPAC, Europeans

## Abstract

**Background:**

Type 2 diabetes (T2D) is a heterogeneous disease with well-known genetic and environmental risk factors contributing to its prevalence. Epigenetic mechanisms related to changes in DNA methylation (DNAm), may also contribute to T2D risk, but larger studies are required to discover novel markers, and to confirm existing ones.

**Results:**

We performed a large meta-analysis of individual epigenome-wide association studies (EWAS) of prevalent T2D conducted in four European studies using peripheral blood DNAm. Analysis of differentially methylated regions (DMR) was also undertaken, based on the meta-analysis results. We found three novel CpGs associated with prevalent T2D in Europeans at cg00144180 (*HDAC4*), cg16765088 (near *SYNM*) and cg24704287 (near *MIR23A*) and confirmed three CpGs previously identified (mapping to *TXNIP*, *ABCG1* and *CPT1A*). We also identified 77 T2D associated DMRs, most of them hypomethylated in T2D cases versus controls. In adjusted regressions among diabetic-free participants in ALSPAC, we found that all six CpGs identified in the meta-EWAS were associated with white cell-types. We estimated that these six CpGs captured 11% of the variation in T2D, which was similar to the variation explained by the model including only the common risk factors of BMI, sex, age and smoking (*R*^2^ = 10.6%).

**Conclusions:**

This study identifies novel loci associated with T2D in Europeans. We also demonstrate associations of the same loci with other traits. Future studies should investigate if our findings are generalizable in non-European populations, and potential roles of these epigenetic markers in T2D etiology or in determining long term consequences of T2D.

## Background

Type 2 diabetes (T2D) is a heterogeneous disease with both environmental and genetic factors implicated in disease onset and progression [1, 2]. The contribution of environmental factors (e.g. diet, physical inactivity, smoking and ethnicity) in T2D etiology is well-known [3–8]. Recent large Genome Wide Association Studies (GWAS) have identified > 400 genetic variants associated with T2D that together explain around 15–18% of T2D estimated heritability [9, 10]. However, the pathophysiology linking many of these environmental and genetic factors with disease onset and progression is less well understood. Consequently, there is growing interest in understanding the role of epigenetic mechanisms surrounding T2D [7].

In epidemiological studies, DNA methylation (DNAm) is the most widely studied epigenetic mechanism, partly due to the fact that it can be measured at scale [11–13]. DNAm is a heritable mark associated with regulation of gene expression and high-order DNA structure [14]. Because DNAm can be modified in response to lifestyle and environmental factors [15] and is associated with genetic variants [16–18], the study of disease-related dysregulation in DNAm could ultimately reveal novel mechanisms in the pathophysiology of T2D, provide new drug targets, and facilitate the discovery of prognostic biomarkers in non-invasive tissues such as peripheral blood [15, 19]. Existing evidence demonstrates the association between T2D and DNAm in metabolically relevant tissues [20], and in blood [1, 2, 14, 15, 21]. So far, prospective longitudinal studies have discovered up to 18 blood-based CpG sites associated with future liability for T2D [19, 22, 23]. Many of these CpG sites are also associated with prevalent T2D [1, 2, 14, 21, 23, 24]. However, most of the latter studies have been conducted in relatively modest sample sizes, rarely using meta-analysis to increase sample size and power to detect differential methylation. To reveal new loci (which could be informative of disease onset or progression) and to confirm previously reported associations, we conducted a large meta-analysis of prevalent T2D using epigenome-wide DNAm from blood samples in four European cohorts. We then implemented functional analyses to investigate possible mechanisms explaining the association between identified markers and prevalent T2D.

## Results

### Study characteristics

Four cohorts carried out independent EWAS and provided summary statistics for meta-analysis. These were: The Avon Longitudinal Study of Parents and Children (ALSPAC), The Lothian Birth Cohort of 1936 (LBC1936), and two sub-cohorts of the Rotterdam Study (RSIII-1 and RS-Bios) (Table 1). Of 3,428 total participants, 340 (10%) had diabetes. Individuals with diabetes had on average higher BMI, fasting glucose (or the hemoglobin A1c (HbA1c) in LBC1936), systolic blood-pressure, LDL and triglyceride levels compared with controls (Additional file 1: Table S2). In most cohorts there was little evidence of difference in age, sex and smoking between cases and controls. We identified < 5 (< 0.9%), 37 (11.2%), 41 (6.4%) and 24 (3.9%) new cases of T2D among 558, 329, 643 and 612 controls with available follow-up data in ALSPAC, LBC1936, RSIII-1, and RS-Bios, respectively. New cases were detected after a follow-up period ranging from 5-years (ALSPAC) to 12-years (LBC1936).

**Table 1:** Baseline characteristics of participants in four European cohorts included in the meta-analysis of EWAS of prevalent T2D

Cohort	*N*	*N* T2D cases (%)	Mean age in years (SD)	*N* males (%)	Mean FG in mmol/L (SD)	Mean BMI in kg/m^2^ (SD)	*N* current smokers (%)
ALSPAC	1050	48 (4.6)	49.9 (5.4)	405 (38.6)	5.4 (1.1)	26.8 (4.7)	97 (9.2)
LBC1936	915	110 (12.0)	69.6 (0.8)	462 (50.5)	NA^a^	27.8 (4.4)	103 (11.3)
RSIII-1	728	74 (10.2)	59.7 (8.1)	335 (46.0)	5.5 (1.1)	27.5 (4.7)	196 (26.9)
RS-Bios	735	108 (14.7)	67.6 (6.0)	312 (42.4)	5.7 (1.1)	27.7 (4.1)	77 (10.5)
Total	3428	340	61.7	1514	5.5	27.5	473

Continuous variables were described using the mean and SD, and the frequency and percent for categorical variables

SD, standard deviation; BMI, body mass index

^a^Mean values of fasting glucose (FG) were not available in LBC1936. Instead, mean levels of the hemoglobin A1c or HbA1c (%) was reported for these participants: 5.92% (SD = 0.71)

### T2D is strongly associated with peripheral blood DNA methylation at six CpGs

**Fig. 1:**
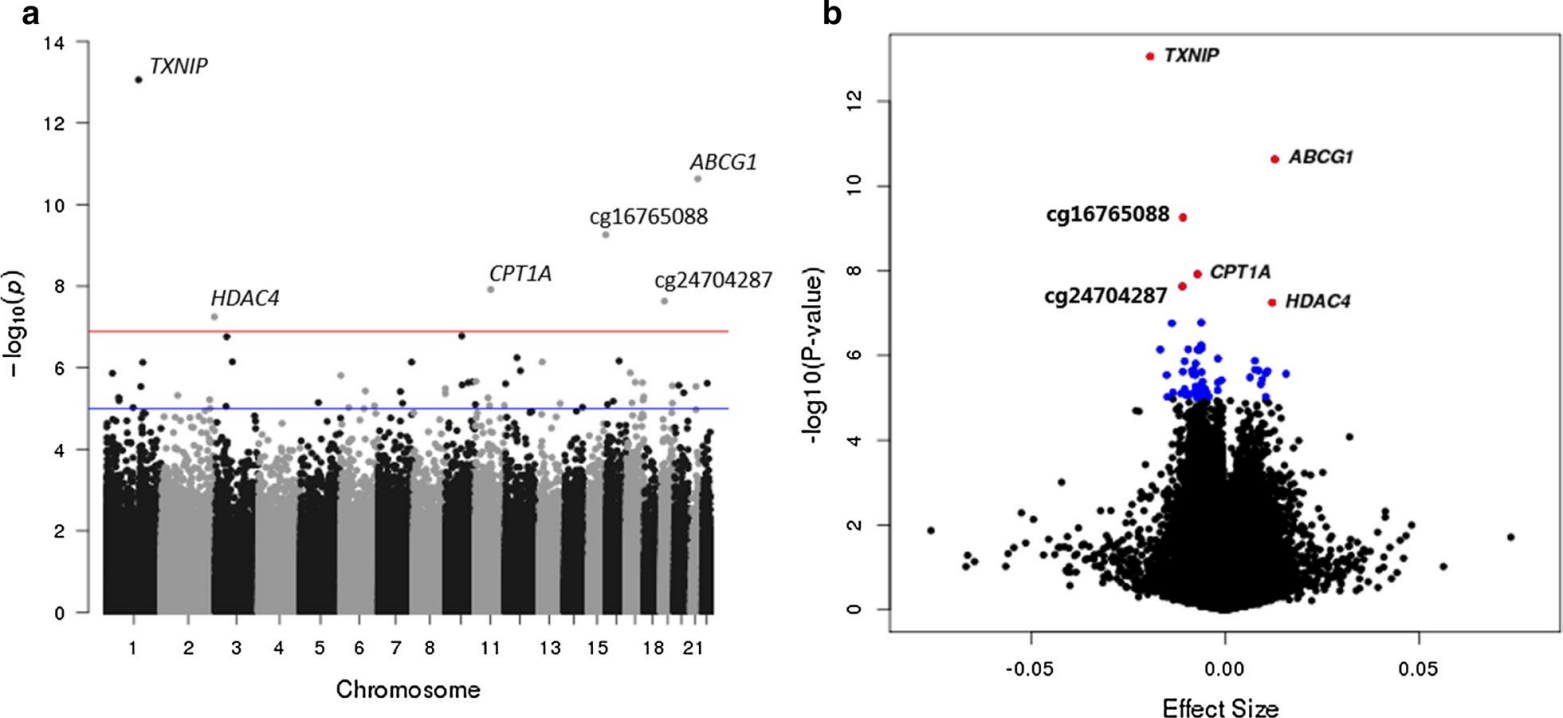
Manhattan (**a**) and volcano plot (**b**) illustrating the association of DNAm in peripheral blood of middle-age and older adults, with prevalent T2D from a meta-analysis with four cohorts: ALSPAC, LBC1936, RSIII-1 and RS-Bios (*n* = 3,428 total; *N* = 340 T2D cases). Results were adjusted for age, sex, SVs, cell counts and smoking. Associations at *p* < 1.33 × 10^–7^ are above the horizontal red line in the Manhattan plot. The horizontal blue line indicates *p* < 1.0 × 10^–5^. The volcano plot shows the distribution of effect sizes against the − log10(*p* value) for all CpG sites. Associations at *p* < 1.33 × 10^–7^ are shown in red and those with *p* < 1.0 × 10^–5^ are highlighted in blue

In the meta-EWAS (model 1, lambda = 1.33), we identified 58 CpGs associated with T2D at *p* < 1.0 × 10^–5^. Six associations were at *p* < 1.3 × 10^–7^ (Table 2). Associations between T2D and CpGs cg11024682 (*SREBF1*) and cg18181703 (*SOCS3*) have been reported previously [22, 23, 25]. The median absolute difference in effect size amongst the top 58 CpGs from the meta-analysis was 0.8% (range 0.1% to 1.9%) (Fig. 1b), or an absolute difference in 0.2 SDs of DNAm (range 0.0002 SDs to 0.14 SDs) between T2D cases and controls (only in ALSPAC). Adjustment for BMI (model 2) attenuated associations detected in the top six CpG sites identified by *p* value (i.e., 67% less significant signals). Only the associations at cg19693031 (*TXNIP*) and cg16765088 (near *SYNM*) remained significant (*p* < 1.3 × 10^–7^) in the BMI model. We observed that among the CpGs attenuated, one was associated with BMI (cg06500161 in *ABCG1*) and another with waist-circumference (cg00574958 in *CPT1A*) in minimally adjusted regressions conducted in ALSPAC (see below). Distribution of DNAm by T2D status for top six CpGs identified in the meta-analysis is presented in the Additional file 1: Figure S1.

**Table 2:** Association estimates for CpG sites at *p* < 1.0 × 10^–5^ identified in the meta-EWAS of T2D with four cohorts (*n* = 3428 total; *N* = 340 T2D cases)

CpG site	Gene	ALSPAC (*N* = 1050)		LBC1936 (*N* = 915)		RSIII-1 (*N* = 728)		RS-Bios (*N* = 735)		Meta-analysis (*N* = 3428)					
		Beta	P	Beta	P	Beta	P	Beta	P	Beta	SE	P	Direction	*I*^2^	*P*_het_
**cg19693031**^b^	***TXNIP***	− 0.022	5.93E−03	− 0.026	3.88E−06	− 0.019	1.86E−05	− 0.015	1.27E−03	− 0.019	2.59E−03	**8.75E−14**	− − − −	**0.00**	**4.55E−01**
**cg06500161**^a,b^	***ABCG1***	0.026	2.68E−04	0.024	5.18E−08	0.010	5.06E−03	0.008	9.90E−03	0.013	1.92E−03	**2.34E−11**	+ + + +	**77.70**	**3.72E−03**
**cg16765088**	***SYNM***	− 0.021	8.47E−03	− 0.014	4.20E−04	− 0.009	2.20E−02	− 0.009	5.43E−05	− 0.011	1.75E−03	**5.50E−10**	− − − −	**0.00**	**4.29E−01**
**cg00574958**^a,b^	***CPT1A***	− 0.005	3.60E−02	− 0.008	6.68E−05	− 0.018	6.19E−06	− 0.004	1.51E−01	− 0.007	1.25E−03	**1.20E−08**	− − − −	**72.20**	**1.29E−02**
**cg24704287**	***MIR23A***	− 0.009	1.41E−01	− 0.012	1.20E−02	− 0.012	6.71E−04	− 0.011	8.31E−04	− 0.011	1.97E−03	**2.34E−08**	− − − −	**0.00**	**9.68E−01**
**cg00144180**	***HDAC4***	0.012	5.61E−02	0.013	5.88E−04	0.019	3.67E−04	0.008	3.94E−02	0.012	2.23E−03	**5.64E−08**	+ + + +	**4.70**	**3.69E−01**
cg04567334	*CDH23*	− 0.001	7.81E−01	− 0.005	5.49E−02	− 0.007	1.89E−03	− 0.007	9.38E−05	− 0.006	1.18E−03	1.67E−07	− − − −	0.00	6.70E−01
cg10584271	*ITIH1*	− 0.007	3.58E−01	− 0.019	8.08E−04	− 0.017	2.92E−04	− 0.010	2.43E−02	− 0.014	2.63E−03	1.73E−07	− − − −	5.60	3.65E−01
cg26270261	*KRT4*	− 0.004	4.35E−01	− 0.011	8.41E−05	− 0.007	1.37E−02	− 0.004	8.07E−03	− 0.006	1.24E−03	5.68E−07	− − − −	31.10	2.25E−01
cg16575444	*CX3CL1*	0.001	7.90E−01	− 0.004	1.29E−01	− 0.006	7.51E−03	− 0.007	2.90E−05	− 0.006	1.22E−03	6.83E−07	+ − − −	0.00	4.54E−01
cg24512093	*ROBO1*	− 0.018	3.57E−02	− 0.009	7.87E−02	− 0.004	3.28E−01	− 0.011	1.06E−05	− 0.010	1.92E−03	7.16E−07	− − − −	16.50	3.09E−01
cg11983038^a^	*Intergenic*	− 0.027	8.44E−04	− 0.003	7.69E−01	− 0.007	2.44E−01	− 0.025	7.05E−06	− 0.017	3.38E−03	7.23E−07	− − − −	65.10	3.53E−02
cg25136644	*ATG9B*	− 0.010	8.33E−02	− 0.007	4.43E−02	− 0.005	2.25E−02	− 0.012	4.52E−05	− 0.007	1.44E−03	7.27E−07	− − − −	34.80	2.04E−01
cg20812370^a^	*PBX1*	− 0.017	2.53E−02	− 0.017	9.03E−05	− 0.003	1.51E−01	− 0.007	5.11E−04	− 0.007	1.34E−03	7.40E−07	− − − −	70.70	1.67E−02
cg24686009	*RAP1B*	− 0.002	3.16E−04	− 0.001	2.82E−02	− 0.003	3.34E−03	0.000	8.43E−01	− 0.002	3.83E−04	1.19E−06	− − − +	0.20	3.91E−01
cg11024682^b^	*SREBF1*	0.007	1.07E−01	0.007	1.95E−02	0.011	1.08E−03	0.006	1.48E−02	0.008	1.59E−03	1.33E−06	+ + + +	0.00	6.94E−01
cg06114363^d^	*ZNF683*	− 0.016	2.27E−02	− 0.012	5.08E−04	− 0.008	7.40E−03	?	?	− 0.010	2.16E−03	1.37E−06	− − − ?	0.00	5.30E−01
cg01963618	*LOC285768*	− 0.010	1.17E−01	− 0.011	5.08E−03	− 0.007	4.91E−02	− 0.007	1.77E−03	− 0.008	1.58E−03	1.55E−06	− − − −	0.00	8.01E−01
cg22680424	*HCCA2*	0.009	2.38E−01	0.013	6.20E−04	0.007	3.18E−02	0.006	8.16E−03	0.008	1.63E−03	2.16E−06	+ + + +	0.00	4.92E−01
cg19876302	*Intergenic*	− 0.020	2.27E−02	− 0.007	1.06E−01	− 0.010	2.21E−03	− 0.006	5.08E−03	− 0.008	1.70E−03	2.22E−06	− − − −	0.00	4.30E−01
cg08857797	*VPS25*	0.002	8.08E−01	0.012	1.09E−02	0.008	6.17E−03	0.008	2.53E−03	0.009	1.84E−03	2.28E−06	+ + + +	0.00	8.13E−01
cg27374726	*Intergenic*	− 0.006	2.35E−01	− 0.015	5.06E−04	− 0.004	2.66E−01	− 0.011	3.93E−04	− 0.009	1.83E−03	2.32E−06	− − − −	42.40	1.57E−01
cg09185884	*KCTD2*	0.011	2.94E−02	0.015	6.40E−02	0.013	4.18E−02	0.010	1.28E−03	0.011	2.31E−03	2.33E−06	+ + + +	0.00	9.19E−01
cg27115863	*Intergenic*	− 0.023	3.50E−03	− 0.006	2.40E−01	− 0.010	2.27E−02	− 0.012	1.23E−03	− 0.011	2.31E−03	2.41E−06	− − − −	13.40	3.25E−01
cg24795867	*WNT5B*	− 0.007	3.59E−01	− 0.002	6.13E−01	− 0.002	3.62E−01	− 0.008	5.51E−07	− 0.006	1.29E−03	2.47E−06	− − − −	43.20	1.52E−01
cg08945443	*ZMYND17*	− 0.006	3.92E−01	0.009	1.63E−01	0.015	6.67E−04	0.012	2.08E−04	0.010	2.23E−03	2.64E−06	− + + +	51.70	1.02E−01
cg06039489	*C20orf26*	0.008	3.03E−01	0.028	2.22E−03	0.010	1.39E−01	0.018	3.43E−04	0.016	3.35E−03	2.71E−06	+ + + +	16.80	3.08E−01
cg08570691	*RPL13AP5*	− 0.012	7.78E−02	− 0.010	3.93E−03	− 0.014	3.64E−04	− 0.004	8.95E−02	− 0.008	1.76E−03	2.78E−06	− − − −	42.60	1.56E−01
cg12593793	*Intergenic*	− 0.012	2.93E−02	− 0.011	1.17E−03	− 0.014	4.03E−04	− 0.003	2.27E−01	− 0.008	1.65E−03	2.90E−06	− − − −	60.50	5.54E−02
cg27037013	*Intergenic*	− 0.027	2.39E−02	− 0.025	7.68E−04	− 0.019	2.20E−03	− 0.007	1.41E−01	− 0.015	3.22E−03	2.90E−06	− − − −	51.90	1.01E−01
cg07212837	*Intergenic*	0.003	5.45E−01	0.008	6.22E−03	0.007	2.72E−02	0.006	1.93E−03	0.006	1.38E−03	3.28E−06	+ + + +	0.00	8.43E−01
cg16192197	*Intergenic*	0.007	4.31E−01	0.005	3.38E−01	0.009	1.75E−02	0.013	5.29E−05	0.010	2.06E−03	3.71E−06	+ + + +	0.00	5.03E−01
cg15560632	*LRCH4*	− 0.001	7.24E−03	− 0.001	3.40E−03	− 0.002	1.02E−02	− 0.001	1.62E−01	− 0.001	2.04E−04	3.83E−06	− − − −	3.50	3.75E−01
cg14003143^a^	*SGK2*	− 0.021	5.65E−04	− 0.007	1.49E−02	− 0.007	8.21E−03	− 0.004	3.85E−02	− 0.006	1.27E−03	4.12E−06	− − − −	62.60	4.54E−02
cg20154947	*PLEC1*	− 0.002	3.91E−04	− 0.002	1.49E−03	− 0.002	1.48E−01	0.000	7.36E−01	− 0.002	4.09E−04	4.34E−06	− − − −	23.40	2.71E−01
cg25741837	*SMYD5*	0.022	4.86E−03	0.007	9.85E−02	0.009	2.33E−02	0.009	4.74E−03	0.009	2.01E−03	4.76E−06	+ + + +	0.00	3.96E−01
cg26766064	*MIR657*	− 0.014	8.56E−02	− 0.010	7.14E−03	− 0.008	1.59E−02	− 0.005	6.10E−03	− 0.007	1.43E−03	5.17E−06	− − − −	0.00	4.12E−01
cg25536676	*DHCR24*	− 0.007	2.52E−01	− 0.012	1.48E−03	− 0.014	3.67E−04	− 0.004	8.16E−02	− 0.008	1.68E−03	5.39E−06	− − − −	54.80	8.42E−02
cg11376147^b^	*SLC43A1*	− 0.008	1.03E−02	− 0.004	5.34E−02	− 0.007	1.94E−02	− 0.005	1.45E−02	− 0.006	1.27E−03	5.43E−06	− − − −	0.00	7.64E−01
cg20316538	*RUFY4*	− 0.008	1.15E−01	− 0.007	7.53E−03	− 0.007	1.39E−02	− 0.004	1.06E−02	− 0.005	1.16E−03	6.11E−06	− − − −	0.00	6.05E−01
cg18181703^c^	*SOCS3*	− 0.024	7.42E−03	− 0.011	2.08E−02	− 0.016	2.85E−04	− 0.004	2.26E−01	− 0.010	2.31E−03	6.20E−06	− − − −	56.50	7.52E−02
cg11851382	*PPAP2B*	− 0.012	2.34E−02	− 0.012	3.79E−03	− 0.005	2.13E−01	− 0.006	5.87E−03	− 0.008	1.69E−03	6.42E−06	− − − −	0.00	4.91E−01
cg00162348	*RNF40*	− 0.002	1.79E−04	− 0.001	5.06E−02	− 0.003	4.86E−02	− 0.001	5.94E−01	− 0.002	4.23E−04	6.64E−06	− − − −	0.00	6.00E−01
cg07184465	*SPZ1*	− 0.012	3.83E−02	− 0.006	6.66E−02	− 0.011	1.40E−04	− 0.004	1.03E−01	− 0.007	1.55E−03	7.18E−06	− − − −	38.20	1.83E−01
cg14284506	*Intergenic*	− 0.009	8.08E−04	− 0.005	1.01E−03	− 0.003	2.51E−01	− 0.002	5.00E−01	− 0.005	1.08E−03	7.31E−06	− − − −	21.90	2.79E−01
cg11252555^a^	*RPL13AP5*	− 0.013	4.69E−02	− 0.010	3.55E−03	− 0.015	1.85E−04	− 0.003	2.03E−01	− 0.008	1.73E−03	7.44E−06	− − − −	63.30	4.25E−02
cg10082515	*Intergenic*	− 0.022	3.20E−03	− 0.006	5.79E−01	− 0.010	7.04E−02	− 0.014	1.56E−03	− 0.013	3.00E−03	7.46E−06	− − − −	0.00	5.05E−01
cg00896068	*Intergenic*	− 0.003	6.50E−01	− 0.011	7.96E−03	− 0.004	3.10E−01	− 0.009	1.79E−04	− 0.008	1.74E−03	7.58E−06	− − − −	0.00	4.82E−01
cg01577083	*Intergenic*	− 0.018	4.64E−03	− 0.012	4.99E−02	− 0.008	5.13E−02	− 0.012	1.44E−02	− 0.011	2.54E−03	7.93E−06	− − − −	0.00	6.21E−01
cg00320980	*Intergenic*	− 0.006	3.44E−01	− 0.007	8.21E−02	− 0.012	2.75E−03	− 0.010	4.24E−03	− 0.009	2.09E−03	7.97E−06	− − − −	0.00	7.44E−01
cg20231084	*Intergenic*	− 0.011	1.12E−01	− 0.008	1.78E−02	− 0.005	6.20E−02	− 0.006	2.49E−03	− 0.006	1.39E−03	8.36E−06	− − − −	0.00	7.96E−01
cg15832662	*RTN3*	− 0.010	1.70E−01	− 0.012	6.91E−03	− 0.012	5.16E−02	− 0.008	5.65E−03	− 0.009	2.08E−03	8.45E−06	− − − −	0.00	8.29E−01
cg13178597	*RGS17*	− 0.018	1.41E−02	− 0.005	4.64E−01	− 0.009	2.36E−02	− 0.011	1.60E−03	− 0.010	2.27E−03	8.57E−06	− − − −	0.00	5.80E−01
cg20116935	*SEMA3B*	− 0.015	3.85E−03	− 0.005	5.45E−02	− 0.006	6.60E−02	− 0.005	6.69E−03	− 0.006	1.38E−03	8.89E−06	− − − −	4.80	3.69E−01
cg00989505	*MIR299*	0.003	5.16E−01	− 0.004	1.00E−01	− 0.006	4.00E−04	− 0.004	6.73E−03	− 0.004	9.41E−04	9.33E−06	+ − − −	14.80	3.18E−01
cg07068382	*MTCH1*	0.000	9.91E−01	0.020	1.38E−04	0.011	1.32E−02	0.007	3.60E−02	0.010	2.37E−03	9.46E−06	− + + +	46.70	1.31E−01
cg14476101^b^	*PHGDH*	− 0.021	4.05E−02	0.000	9.46E−01	− 0.021	2.41E−04	− 0.017	4.72E−03	− 0.015	3.36E−03	9.46E−06	− − − −	52.00	1.00E−01
cg20456243	*SPEG*	− 0.015	6.49E−02	− 0.004	2.74E−01	− 0.010	3.31E−03	− 0.007	3.41E−03	− 0.007	1.65E−03	9.99E−06	− − − −	0.00	4.90E−01

Associations described correspond to the main meta-EWAS model (model 1) adjusted for age, sex, SVs, cellular heterogeneity and smoking. Highlighted in bold are associations at *p* < 1.33 × 10^–7^. Direction of effect for each CpG is indicated for EWAS in ALSPAC, LBC1936, RSIII-1 and RS-Bios, respectively. *I*^2^ is the heterogeneity statistic. High heterogeneity if *I*^2^ > 40% and *P*_het_ < 0.05

^a^CpGs identified with high heterogeneity in the meta-analysis

^b^CpGs discovered in association with incident T2D, and later validated in studies of prevalent T2D by Cardona et al. [23]

^c^CpG identified in association with incident T2D by Chambers et al. [22]

^d^In RS-Bios, estimates of the EWAS for the CpG cg06114363 (ZNF683) did not surpass QC prior to meta-analysis

**Fig. 2:**
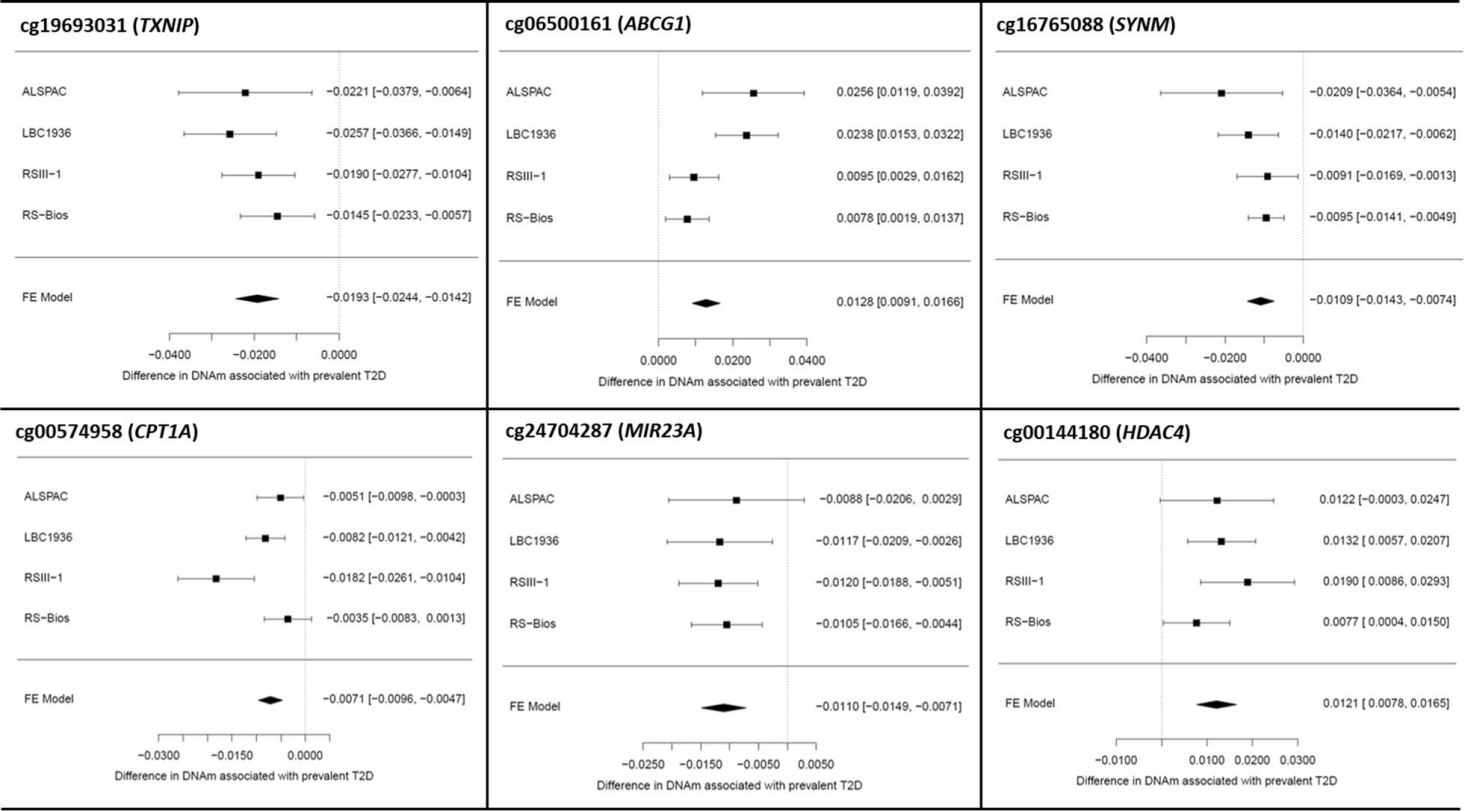
Forest-plots showing results of the meta-EWAS of T2D across four European cohorts for six differentially methylated CpGs identified at *p* < 1.33 × 10^–7^. For each CpG site we show association estimates (effect-size and 95% confidence interval) of the EWAS conducted by each cohort, and the combined estimate using a fixed-effect inverse-variance weighted meta-analysis (diamond at the bottom). Results were adjusted for age, sex, SVs, cellular heterogeneity and smoking

We assessed robustness of our findings to interstudy heterogeneity using different strategies. First, we looked at the *I*^2^ statistic, which showed weak heterogeneity with *I*^2^ < 40% in 4/6 T2D-associated CpG sites in the meta-analysis. Heterogeneity was high for CpGs in *CPT1A* and *ABCG1* (*I*^2^ > 70%, *p* < 0.05) (Table 2). Second, we used a random-effect meta-analysis adjusted for same covariates as in model 1, finding small changes in the effect estimate compared to the fixed-effect analysis at the T2D-associated CpGs (absolute change in effect range: 0% to 21%) (Additional file 1: Table S4). In total, 4/6 differentially methylated CpGs remained associated with T2D at *p* < 1.3 × 10^–7^ in the random-effect analysis: cg19693031 (*TXNIP*), cg00144180 (*HDAC4*), cg16765088 (near *SYNM*) and cg24704287 (near *MIR23A*). Finally, forest-plots showed consistency in the direction of association between studies at our six differentially methylated CpGs (see Fig. 2), and the leave-one-out analysis revealed that no single study was consistently having a disproportionately large influence in the combined effect at these sites (Additional file 1: Figure S2).

### DMR analyses

We identified 77 regions associated with T2D based on the main meta-analysis (Additional file 1: Table S5). Among these regions, we found an overrepresentation for hypomethylated DMRs (*n* = 55/77 DMRs) in association with T2D. The DMR with the smallest Sidak-corrected *p* value overlapping a meta-EWAS signal was identified in an intron of *CPT1A* (estimate = − 0.01, Sidak *p* = 1.11 × 10^–9^). This DMR showed lower methylation values in T2D cases compared with controls. In addition, several DMRs mapped to loci or included CpGs that have been previously associated with prevalent or incident T2D: cg21766592 in *SLC1A5* [2], cg14476101 in *PHGDH* [23] and *PFKB3* [23]. Our findings are directionally consistent with these studies.

### Association between DNAm and phenotypic traits in ALSPAC at key CpGs identified in EWAS meta-analysis

T2D-associated CpG sites were also associated (at *α* = 0.05/23 traits analyzed or *p* < 2.0 × 10^–3^) with age (*HDAC4* & *SYNM*), sex (*TXNIP*, *HDAC4*, *CPT1A*, *SYNM* & *ABCG1*), categories of glucose tolerance (*TXNIP*, *HDAC4*, *CPT1A* & *ABCG1*), fasting insulin and the homeostasis model assessment (HOMA) scores (*ABCG1*), waist-circumference (*CPT1A* & *ABCG1*), BMI (*ABCG1*), C-reactive protein (CRP) (*ABCG1* & *HDAC4*), triglyceride levels (*CPT1A* & *ABCG1*) and HDL levels (*ABCG1*) based on minimally adjusted regressions conducted among diabetes-free participants in ALSPAC (Additional file 1: Table S6). All six CpGs from the meta-analysis were also associated with white cell-types, and the CpG in *MIR23A* was exclusively associated with cell-types and not with clinical phenotypes. Associations with the traits remained directionally consistent in the analysis using DNAm stratified by quartiles (Additional file 1: Table S7–Table S12). Overall, we observed that the six T2D-associated CpGs from the meta-analysis showed directionally concordant associations with fasting glucose, fasting insulin, 2-h glucose and HOMA scores among non-diabetic control participants in ALSPAC (Table 2, Additional file 1: Table S6).

Odds of T2D in the ALSPAC cohort were calculated for the six CpG sites and one DMR identified in the meta-analysis (Table 3). We observed consistency in the direction and strength of associations in ALSPAC compared to results of the meta-analysis. Using the Nagelkerke’s *R*^2^ statistic, we identified small variation in T2D captured by the individual CpG sites (*R*^2^ range 1.3% to 5.7%) or by CpG sites within the DMR. Combining the six differentially methylated CpG sites, we explained 11% of the total variation in T2D using ALSPAC data. This was similar in LBC1936, where combining the six differentially methylated CpG sites explained 13.6% of the total variation in T2D. For comparison, variation attributed to the combined common risk factors of age, sex, BMI and smoking was *R*^2^ = 10.6% in ALSPAC and 12.2% in LBC1936. Variation attributed to these common risk factors and methylation at six differentially methylated CpG sites combined was *R*^2^ = 22% in ALSPAC and 21.3% in LBC1936. Adding fasting glucose to the predictive model with clinical risk factors and CpG sites captured 68% of the variation in T2D in ALSPAC. We could not perform the same estimation in LBC1936 due to lack of fasting glucose measures and high collinearity between HbA1c, the available glucose trait in this cohort, and T2D.

**Table 3:** Odds of T2D for six CpG sites and one DMR identified in the meta-EWAS of T2D

CpG	Chr	Gene	Genomic Feature	Main model			BMI-adjusted model			Variation in T2D (%)^a^
				OR	95% CI	*P*	OR	95% CI	*P*	
**cg19693031**	**1**	***TXNIP***	3′UTR	**0.93**	**(0.89,0.98)**	**1.10E−02**	**0.94**	**(0.89,0.99)**	**3.00E−02**	2.0
**cg00144180**	**2**	***HDAC4***	5′UTR	**1.08**	**(1.01,1.16)**	**2.40E−02**	**1.08**	**(1.00,1.17)**	**4.00E−02**	2.9
cg00574958	11	*CPT1A*	5′UTR	0.79	(0.62,1.00)	5.13E−02	0.83	(0.65,1.04)	1.10E−01	1.3
**cg16765088**	**15**	***SYNM***	Intergenic	**0.93**	**(0.88,0.99)**	**1.64E−02**	**0.93**	**(0.88,0.99)**	**2.00E−02**	3.3
cg24704287	19	*MIR23A*	Intergenic	0.95	(0.89,1.02)	1.57E−01	0.96	(0.89,1.03)	2.20E−01	1.3
**cg06500161**	**21**	***ABCG1***	Body	**1.13**	**(1.06,1.21)**	**3.77E−04**	**1.1**	**(1.03,1.18)**	**1.00E−02**	5.7
DMR	11	*CPT1A*	Intron	**0.65**	**(0.45,0.92)**	**1.42E−02**	0.7	(0.48,1.00)	5.00E−02	1.1

Highlighted in bold are associations with *p* < 0.05. Associations were conducted in a subsample of ALSPAC (*n* = 1050, *N* = 48 T2D cases). Main model was adjusted for age, sex, SVs, 6-Houseman cells and smoking, and a second model was additionally adjusted for BMI

^a^Variation calculated using the Nagelkerke’s *R*^2^ statistic derived from an unadjusted logistic regression

### Pathway enrichment and cross-tissue comparison of DNAm at CpG sites and DMRs associated with T2D

We ran pathway analyses using a total of 80 unique CpG sites, including the six T2D-associated CpGs from the meta-analysis, and 77 index CpGs with the smallest *p* value identified within each DMR identified. After applying correction for multiple testing, none of the GO terms or KEGG pathways [26] were enriched (Additional file 1: Table S13). For the *in-silico* comparison of DNAm across tissues, we observed positive correlations between DNAm levels in blood cells and five tissues (r range 0.81 to 0.97, *p* < 0.05) at the six identified CpGs in the meta-analysis (Additional file 1: Table S14), and at the 80 CpG sites identified across analyses (Additional file 1: Figure S3). In the enrichment analysis using LOLA [27], we did not identify regulatory elements overlapping with the genomic position of hyper- (*n* = 23 out of 80) or hypomethylated sets (*n* = 57 out of 80) associated with T2D.

**Fig. 3:**
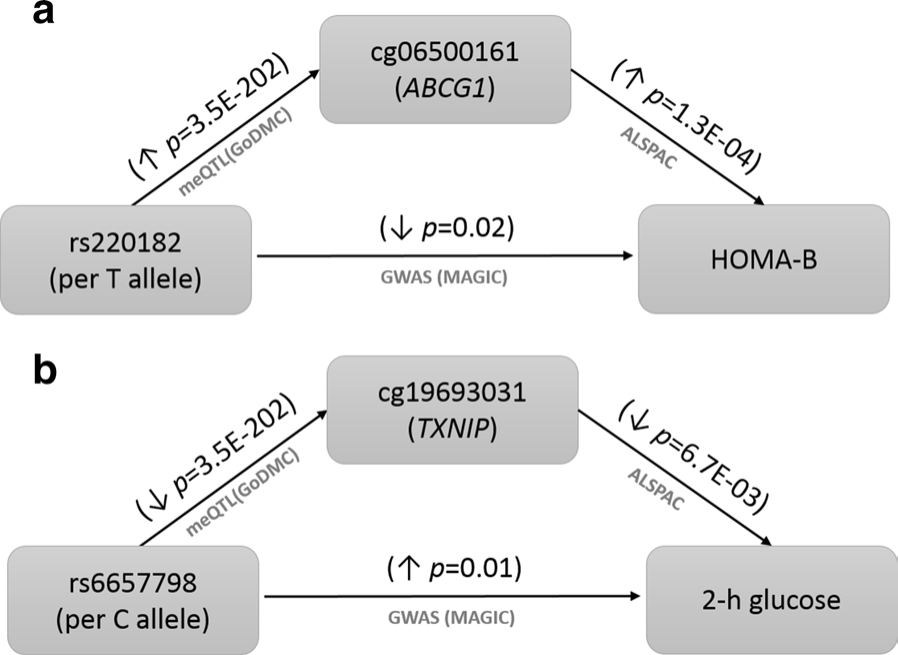
Overlap of a cis-meQTL for cg06500161 (ABCG1, SNP rs220182) and a trans-meQTL for cg19693031 (TXNIP, SNP rs6657798), with **a** a GWAS SNP for HOMA-B and **b** a GWAS SNP for 2-h glucose, respectively. meQTL were retrieved from the Genetics of DNAm consortium (GoDMC, www.godmc.org.uk/) [29] at *p* < 10^–8^ for cis-meQTL (SNPs within 1 Mb from CpG position) and at *p* < 10^–14^ for trans-meQTL (SNPs > 1 Mb or in different chromosomes from CpG position). GWAS SNPs for the glycemic traits were retrieved from the MAGIC consortium (https://www.magicinvestigators.org/) [30, 31]. Associations of peripheral blood DNAm with HOMA-B and 2-h glucose were estimated using linear regressions adjusted for age and sex, when appropriate, in two subsamples of diabetes-free individuals in ALSPAC (*n* = 622 for HOMA-B (only females) and *n* = 1002 for 2-h glucose)

Information from publicly available resources revealed different molecular markers associated with some of our six differentially methylated CpGs from the meta-analysis. Using the BIOS QTL browser [28], we identified expression quantitative trait methylation sites (eQTMs) (at FDR < 0.05) for CpG sites in cg19693031 (*TXNIP*), cg06500161 (*ABCG1*) and cg00574958 (*CPT1A*), where peripheral blood DNAm was inversely associated with gene expression in blood of specific transcripts within the same genes (Additional file 1: Table S15). Using data from the Genetics of DNAm consortium (GoDMC) [29], we found six methylation quantitative trait loci (meQTL) in blood (five in *cis* and one in *trans*) associated with five of our six T2D-associated CpGs in cg19693031 (*TXNIP*), cg06500161 (*ABCG1*), cg00144180 (*HDAC4*) (*n* = 2 meQTL), cg16765088 (*SYNM*) and cg24704287 (*MIR23A*) (Additional file 1: Table S16). Using GWAS data, we found weak evidence that the *trans* meQTL rs6657798 for *TXNIP* (*p* = 3.5 × 10^–202^) was associated with 2-h glucose [30], and that the *cis* meQTL rs220182 for *ABCG1* (*p* = 3.5 × 10^–202^) was associated with the homeostasis model assessments for β-cell function (HOMA-B) [31] (Fig. 3, Additional file 1: Table S17). For the SNP identified in common, the same effect allele had opposite effects on DNAm and on the glycemic trait. In both cases, associations between SNPs and traits were observed with unadjusted association *p* < 0.05. Additionally, no formal causal analysis or colocalization method was applied to establish if an association was present between DNAm at the CpGs and the glucose trait due to a shared causal variant. While of interest, these findings therefore need to be interpreted with caution.

## Discussion

Our meta-EWAS of prevalent T2D achieves a sample size of more than double that of previous studies [1, 14]. Even though the proportion of cases in our study was relatively low (10%), it was consistent with the percent prevalence of T2D reported in other population-based European studies (i.e., 4.6–16% prevalence) [1, 14]. In this study, we found that prevalent T2D was associated with blood DNAm at six individual CpGs in the EWAS and at 77 genomic regions in the DMR analysis, some of them mapping to loci or including CpGs identified in previous studies of prevalent or incident T2D. For CpGs that have been reported in other studies that were not replicated in our meta-analysis, we found that hypermethylation in cg11024682 (*SREBF1*) and hypomethylation in cg11376147 (*SLC43A1*), cg18181703 (*SOCS3*) and cg14476101 (*PHGDH*), were nominally associated with T2D cases versus controls, in agreement with the original studies [22, 23, 25]. Additional analyses in the ALSPAC cohort revealed that most of our top six sites from the meta-analysis were also associated with categories of glucose tolerance, age, sex, white cell-types and other clinical phenotypes. Finally, we interrogated publicly available databases to investigate the potential functional role of the CpG sites identified. We observed strong correlation between DNAm levels in blood and in other tissues of relevance for T2D. In addition, we found meQTL, eQTMs and GWAS SNPs for HOMA-B and 2-h glucose related to some of the CpGs detected in the meta-analysis.

For the strongest meta-EWAs association at *TXNIP*, T2D cases had on average 1.6% lower DNAm levels compared with controls, which represents approximately 3.2% of the overall variance in methylation observed at this site (*TXNIP* DNAm β-values range: 50–100%). Direction of the association at *TXNIP* was consistent with previous findings [2, 14, 22–25], but the effect size we identified was slightly smaller than previously reported (effect range between − 3 and − 5%) [14, 23–25]. In follow-up analyses in ALSPAC, we demonstrated that per 1% increase in methylation at *TXNIP* was associated with 6% lower risk of prevalent T2D. This effect was similar to that reported by Chambers et al*.*[22] for incident T2D. Furthermore, we observed that with respect to controls, participants with diabetes in ALSPAC had 1.2% lower DNAm at *TXNIP* compared to those with prediabetes. Overall, our results at *TXNIP* are consistent with previous studies suggesting that hypomethylation of this CpG could be a good prognostic marker for T2D as well as indicating prevalent disease [1, 14].

DNAm at cg06500161 annotated at *ABCG1* was associated with T2D but not independently of BMI. DNAm at *ABCG1* was higher in T2D cases compared with controls, which is consistent with previous evidence at this locus in blood [2, 19, 22], and in adipose tissue of monozygotic twins discordant for T2D [19, 23]. Among diabetes-free participants in ALSPAC, *ABCG1* was positively associated with BMI, waist-circumference, fasting insulin, HOMA scores, CRP and triglyceride levels, but negatively associated with HDL levels. Direction of association of *ABCG1* methylation with anthropometric [32], glycemic traits [19, 33–36], HDL [34] and triglyceride levels [19, 34, 35] was consistent with previous findings in blood. Thus, differences in *ABCG1* methylation may appear before T2D, but due to our cross-sectional study design we can’t confirm true direction of this association. The lookup in the BIOS QTL browser revealed that DNAm at *ABCG1* was inversely correlated with gene expression of the same gene, and this finding agrees with previous observations in blood [22, 34–36] and in primary target tissues for T2D [22], suggesting that expression of *ABCG1* may be under epigenetic control. No genetic variants predisposing for T2D have been previously reported at *ABCG1* in GWAS studies [37]. However, Hidalgo et al*.*[33] previously identified an association between a *cis* meQTL for *ABCG1* and fasting insulin and the homeostasis model assessment for insulin resistance (HOMA-IR). In our study, we reported a nominal association between a *cis* meQTL for *ABCG1* retrieved from GoDMC, and HOMA-B at GWAS *p* value < 0.05 (unadjusted *p* value).

Lower DNAm at cg00574958 in *CPT1A* was associated with T2D, and this association also appeared to be dependent on BMI. Analyses in ALSPAC showed that per 1% increase in methylation at *CPT1A* was weakly associated with 21% lower risk of T2D. Direction of the association at *CPT1A* methylation was consistent with previous findings at this locus [23, 24]. We also showed that *CPT1A* methylation was inversely associated with waist-circumference and triglycerides levels in the continuous analysis, and with BMI, fasting insulin, HOMA scores and CRP in the stratified analysis by quartiles of DNAm using control samples in ALSPAC. Associations of *CPT1A* with anthropometric traits [32, 38] and triglyceride levels [39] were directionally consistent with previous studies in blood. Other associations with cg00574958 in *CPT1A* have been identified in EWAS of fasting blood lipids [39–41], adiponectin [42], the metabolic syndrome[43], and cardiovascular disease (CVD) risk [44]. Using the BIOS QTL browser, we identified an eQTM in blood associated with cg00574958 in *CPT1A*, indicating that DNAm at this site was inversely associated with expression of a transcript in the same gene, in line with findings by Irvin et al*.*[39]. *CPT1A* encodes for the carnitine palmitoyl-transferase 1A enzyme, a protein essential in the oxidative metabolism of fatty acids in the mitochondrion, and in the secretion of glucagon in pancreatic islets [45]. Some evidence of a causal effect of DNAm at *CPT1A* on T2D was recently demonstrated by Cardona et al*.*[23] using a Mendelian randomization analysis. However, further evidence is required to validate this result.

We reported three novel CpG sites associated with T2D: cg00144180 (*HDAC4*), and two intergenic CpGs in cg16765088 (near *SYNM*) and cg24704287 (near the micro RNA *MIR23A*). However, the role of these CpG sites in T2D is yet unknown. In disease-free individuals in ALSPAC, these CpGs were also associated with CRP (*HDAC4*), and with cell-types (*HDAC4, MIR23A* and *SYNM*), suggesting a potential role of DNAm at these sites in T2D through inflammatory pathways. The CpG cg24704287 near *MIR23A* was exclusively associated with cell-types, indicating that this site may be specifically tagging differences in cell composition between T2D cases and controls. In addition, other EWAS have identified an association between *HDAC4* and BMI [38], and between cg24704287 (*MIR23A*) and levels of the soluble Tumor Necrosis Factor Receptor-2 [46] and smoking [47]. Histone deacetylases (HDACs) are enzymes that catalyze the removal of acetyl groups from lysine residues of non-histone and histone proteins, facilitating gene transcription [48]. Animal models have shown that overexpression of *HDAC4* causes reduction of β-cell mass, and ongoing clinical trials are evaluating the utility of inhibitors and activators of HDACs in T2D therapy [48]. We also observed weaker associations between T2D and cg11024682 (*SREBF1*), cg18181703 (*SOCS3*), cg14476101 (*PHGDH*) and cg11376147 (*SLC43A1*) at *p* values in the order of 10^–5^, helping to confirm previous reports of these CpG sites in studies of incident and prevalent T2D [22, 23].

The enrichment analysis did not uncover any potential novel pathways or gene regulatory elements related with T2D-associated hypo- or hypermethylated sets. An *in-silico* analysis demonstrated high correlation between methylation in blood and five internal target tissues based on CpGs identified in the meta-EWAS (*n* = 6 sites) and the DMR analysis (*n* = 77 index CpGs). Therefore, it is unclear whether differences in DNA methylation identified from blood cells are biologically relevant to T2D disease processes, but patterns in blood do appear to represent those seen in disease relevant tissues. Overall, CpG sites identified offer utility in improving our understanding of underlying disease mechanisms. Further validation of these sites in prospective studies will examine them as predictive markers of incident disease, or of adverse complications of T2D.

Results of the model with additional adjustment for BMI revealed a decrease in the effect size and increase in model *p* value for most of the associations identified in the meta-analysis, but not for associations at cg19693031 (*TXNIP*) and cg16765088 (near *SYNM*). Thus, for the remaining CpGs, the association with T2D may have been influenced by underlying differences in BMI between T2D cases and controls. By using a reverse analysis, we were able to estimate the proportion of variation in T2D explained by CpGs in the meta-analysis, which was similar in magnitude to the variation attributed to the common risk factor of age, sex, BMI & smoking, but much lower than that considering fasting glucose as a predictor. In combination, CpGs and clinical risk factors explained up to 68% of the variation in T2D. However, these results should be cautiously interpreted as variation in T2D attributed to the discovered CpG sites was estimated in two cohorts included within the meta-analysis.

### Strengths and limitations

Our study benefits from the large sample-size included in the meta-analysis, which allowed us to confirm previous associations with T2D and to report novel findings. By establishing an analysis plan shared across cohorts, we were able to minimize potential sources of technical noise in the data. In addition, we conducted different exploratory methods to test for robustness of our findings to inter-study heterogeneity and reported the association of our top signals with metabolic and anthropometric traits of relevance in T2D. Furthermore, the use of blood as the source of DNAm markers allowed us to identify novel non-invasive signatures of prevalent disease. Future research should explore the application of these signals in T2D incidence, prognosis and monitoring of complications, irrespective of their role in T2D etiology [49]. One of the limitations of this study was the use of a cross-sectional study design, meaning that we cannot establish if observed variation in methylation occurred as a cause or a consequence of T2D. Thus, further studies using a longitudinal approach to assess incident T2D, or implementing causal inference methods such as Mendelian randomization [50–52], are required to establish true direction of causality in the association between DNAm and T2D. Another limitation was the use of samples included in the main analysis to estimate proportion of variation in T2D explained by the identified CpGs, which could give biased results. Ideally, T2D variation should be estimated in an independent sample using a weighted methylation score to assess in combination, the predictive ability of the identified markers. Finally, because our sample was restricted to participants of European origin, the generalizability of our novel markers to other populations is still unknown.

## Conclusions

We detected cross-sectional associations between blood DNAm and T2D that were consistent with results in previous studies of incident and prevalent T2D in participants of European and non-European ancestry. Novel markers were also identified. Assessment of the etiological role of markers reported in this study may benefit from the use of causal inference methods such as Mendelian randomization, and from the incorporation of tissue-specific analyses. CpG sites and regions identified in this study could potentially be used as prognostic biomarkers for disease onset or complications.

## Materials and methods

### Study population

Four European cohorts were included: the Avon Longitudinal Study of Parents and Children (ALSPAC) (*N* = 1,050, *n*_T2D_ = 48) [53, 54]; the Lothian Birth Cohort of 1936 (LBC1936) (*N* = 915, *n*_T2D_ = 110) [55–57]; and two independent samples from the Rotterdam Study: RSIII-1 (*N* = 728, *n*_T2D_ = 74) and RS-Bios (*N* = 735, *n*_T2D_ = 108) [58, 59]. In all cohorts, participants provided written informed consent at enrollment, and ethical approval was granted by the relevant ethics and law committees [53, 56, 58, 60]. Further description of the participating cohorts in our meta-analysis can be found in the Additional file 1: section I.

### Phenotypic measurement

Prevalent T2D was defined based on medical diagnosis, use of medication to lower blood glucose, fasting blood glucose levels > 7.0 mmol/l or hemoglobin A1c (HbA1c) levels > 6.5%. Further detail of case ascertainment in each study is described in the Additional file 1: section I. Controls were participants without medical diagnosis of T2D, fasting blood glucose < 7.0 mmol/L, HbA1c < 6.5% (when available), and no reported use of glucose-lowering drugs. Age, sex and smoking were extracted from questionnaire data. Missing values for smoking in one of the cohorts were predicted using a methylation score (Additional file 1: section II). Body mass index (BMI) was calculated from study clinic assessed weight and height (kg/m^2^).

### DNA methylation measurement

As far as possible, cohorts followed a uniform procedure to generate data. Briefly, purified DNA from whole blood samples was bisulfite converted using the Zymo EZ DNA Methylation™ kit (Zymo Research Corporation, Irvine, USA) and hybridized to the Illumina Infinium HumanMethylation 450 BeadChip (HM450) (Illumina, CA, USA). Samples were excluded if signal detection rate across probes was < 95%. Further detail of quality control steps applied to the methylation data at the probe and sample level, are described in the Additional file 1: section III, Table S1. To control for differences in DNAm arising from cellular heterogeneity, cell proportions for six leucocyte subtypes were calculated from DNAm data [61]. In RS-Bios, direct counts for lymphocytes, monocytes and granulocytes were initially available for all participants. Using directly measured cell counts instead of Houseman cell count estimates in RS-Bios did not impact on the results observed. Cohorts corrected for batch effects using surrogate variable (SV) analysis [62]. Ten SVs were included as covariates in the EWAS provided they were individually independent of T2D status (*p* > 0.05). Lastly, samples with extreme measures of DNAm were identified using the Tukey method [63] and set to missing values before conducting the EWAS. This latter step involved trimming DNA methylation beta values for each CpG site by removing observations that were more than three times the interquartile range below or above the 25^th^ and 75^th^ percentiles, respectively [64]. This method allows to exclude outliers of DNA methylation caused by technical artefacts or rare genetic variants that can bias the analysis [65]. This method has been widely implemented in other studies [65–67].

### Statistical analyses

#### Epigenome-wide association study of prevalent T2D

Each cohort conducted independent EWAS using the *meffil* R package [68], where DNAm was modeled as the outcome and T2D as the exposure in multivariable linear regression models. The basic model (model 1) was adjusted for age, sex, SVs, cell counts and smoking status, in model 2 we additionally included BMI. The genomic-inflation factor (*λ*) was used to report systematic bias in EWAS results. Before meta-analysis, EWAS estimates were inspected for potential sources of bias using the *QCEWAS* R package [69] (Additional file 1: section IV). High quality probes obtained in the standardized QC process were included in a fixed-effect inverse variance weighted meta-analysis in METAL (version 2011-03-25) [70]. On average, 368,208 autosomal probes (probe range 349,413 to 376,820) were meta-analyzed. We applied correction for multiple testing using the Bonferroni method, considering associations at *p* value < 1.33 × 10^–7^. Heterogeneity was evaluated using the *I*^2^ statistic (substantial heterogeneity was defined as *I*^2^ > 40% and heterogeneity *p* < 0.05), and by visualizing results in forest plots and conducting “leave-one-out” analyses using the *metafor* R package [71]. To further assess robustness of our findings to inter-study heterogeneity, we conducted a random-effect meta-analysis using a modified version of METAL [70].

CpGs with *p* values below an arbitrary *p*-threshold < 1.0 × 10^–5^ were reported. For these CpGs, effect estimates were described using the median absolute difference in methylation β-values between T2D cases and controls. Furthermore, we re-ran analyses for these CpGs using *z*-values of methylation (mean = 0 and SD = 1) based on data from the ALSPAC study to provide effect estimates standardized by baseline differences in DNAm. Standardized effects were described as the median absolute difference in standard deviations (SDs) of DNAm between groups. We investigated DMR associations with prevalent T2D using *comb-p* [72] based on summary statistics from the meta-analysis, and using default analysis parameters. All analyses were conducted in R (version 3.3.3) [73].

#### Association of DNAm with phenotypic traits in ALSPAC

Additional analyses in ALSPAC investigated whether the individual CpG sites identified in the meta-analysis (at *p* < 1.33 × 10^–7^) were associated with various clinical phenotypes (Additional file 1: section V). We tested associations using multivariable linear and logistic regressions adjusted for age and sex, where DNAm at the identified CpGs was modelled as the exposure (continuous) against the traits. Analyses were restricted to diabetes-free participants to avoid bias by T2D treatment. We repeated analyses stratifying methylation at each CpG into quartiles to assess robustness of associations. We applied Bonferroni adjustment for multiple testing assuming 23 independent tests (i.e. number of different traits) and *α* = 0.05. Furthermore, we investigated the proportion of variation in T2D explained by CpG sites discovered in the meta-analysis (*p* < 1.33 × 10^–7^), and by CpGs within the single DMR with the smallest corrected *p* value, using the Nagelkerke’s *R*^2^ statistic. This statistic was derived from adjusted logistic regressions between DNAm at the individual CpGs in the meta-EWAS or the average methylation at CpGs within the top DMR as the exposure, and T2D as the outcome (reverse model from the EWAS, Additional file 1: section V).

#### Pathway enrichment and cross-tissue comparison of DNAm at CpGs identified in the meta-analysis

We conducted pathway analyses using CpGs identified in the meta-analysis (*p* < 1.33 × 10^–7^), and the CpG with the smallest *p* value identified in each DMR (Sidak *p* value < 0.05). We performed an enrichment analysis for biological pathways using gene ontology (GO) terms and KEGG pathways implemented in the *missMethyl* R package [26]. *p* values of enrichment were adjusted for multiple testing using the Bonferroni method (i.e. *p* < 0.05/# genes in a pathway). For the same list of CpGs, we compared the level of DNAm between blood and five internal tissues relevant to diabetes (liver, skeletal muscle, pancreas, visceral fat or omentum and subcutaneous fat) using a publicly available dataset [74]. We performed an enrichment analysis for regulatory elements using LOLA [27] and identified expression quantitative trait methylation sites (eQTMs) and methylation quantitative trait loci (meQTL) associated with our CpGs from public databases (BIOS QTL browser [28], GoDMC [29]). In addition, we looked up blood-based meQTL associated with our CpGs in expression quantitative trait loci (eQTL) data [75], and in GWAS for T2D [76] and glycemic traits [30, 31, 77, 78] (Additional file 1: section VI) to understand the possible function of the identified CpGs.

## Supplementary Information

**Additional file 1**. Additional information supporting findings of the present study.

## Data Availability

The datasets used during the current study are available from the corresponding author on reasonable request.

## Abbreviations

ALSPAC: Avon Longitudinal Study of Parents and Children
BMI: Body mass index
CpG: Cytosine-phosphate-guanine
CRP: C-reactive protein
CVD: Cardiovascular disease
DBP: Diastolic blood-pressure
DMR: Differentially methylated region
DNAm: DNA methylation
eQTMs: Expression quantitative trait methylation sites
EWAS: Epigenome-wide association studies
FDR: False discovery rate
FG: Fasting glucose
GO: Gene ontology terms
GoDMC: The genetics of DNAm consortium
GWAS: Genome Wide Association Studies
HbA1c: Hemoglobin A1c
HDACs: Histone deacetylases
HOMA-IR: Homeostasis model assessment for insulin resistance.
HOMA-B: Homeostasis model assessment for β-cell function
KEGG: Kyoto Encyclopedia of Genes and Genomes
LBC1936: Lothian Birth Cohort of 1936
LDL: Low-density lipoproteins
LOLA: Locus Overlap Analysis for Enrichment of Genomic Regions
meQTL: Methylation quantitative trait loci
Meta-EWAS: Meta-analysis of individual EWAS
RSIII-1: Sub-cohort of the Rotterdam Study consisting of participants from the first visit of the third Rotterdam cohort
RS-Bios: Sub-cohort of the Rotterdam Study consisting of participants from the third visit of the second Rotterdam cohort (RSII-3), and those from the second visit of the third cohort (RSIII-2), non-overlapping with participants from RSIII-1
SBP: Systolic blood-pressure
SD: Standard deviation
SV: Surrogate variable
T2D: Type 2 diabetes
VLDL: Very low-density lipoproteins
