# Supplementary material for: DNA methylation of blood cells is associated with prevalent type 2 diabetes in a meta-analysis of four European cohorts
Source: Clin Epigenetics. 2021 Feb 23;13:40. doi: 10.1186/s13148-021-01027-3 (PMC7903628; doi:10.1186/s13148-021-01027-3)
Supplement: Supplementary file 1 — Additional file 1. Additional information supporting findings of the present study. [file 13148_2021_1027_MOESM1_ESM.docx]

**Supplementary Material**

**Table of Contents**

[I. Cohort Description 3](#_Toc46410850)

[The Avon Longitudinal Study of Parents and Children (ALSPAC) 3](#_Toc46410851)

[The Lothian Birth Cohort of 1936 (LBC1936) 4](#_Toc46410852)

[The Rotterdam Study (RS) 4](#_Toc46410853)

[II. Phenotypic measurement 5](#_Toc46410854)

[III. Methylation measurement and quality control 6](#_Toc46410855)

[IV. Quality control of individual EWAS results before meta-analysis 7](#_Toc46410856)

[V. Post-hoc analyses implemented in ALSPAC 8](#_Toc46410857)

[VI. Enrichment analysis for regulatory elements and look-up in publicly available databases 9](#_Toc46410858)

[Enrichment analysis for regulatory elements 9](#_Toc46410859)

[eQTMs 9](#_Toc46410860)

[meQTL 10](#_Toc46410861)

[Overlap between meQTL of interest and eQTL in specific tissues 10](#_Toc46410862)

[Overlap between meQTL of interest and GWAS SNPs for T2D and glycemic traits 11](#_Toc46410863)

[VII. References 12](#_Toc46410864)

[List of Figures and Tables 15](#_Toc46410865)

[Figure S1. Distribution of DNA methylation β-values by T2D status for six CpGs identified in the meta-analysis of EWAS 15](#_Toc46410866)

[Figure S2. Leave-one-out for six CpG sites identified in association with T2D in the meta-analysis 16](#_Toc46410867)

[Figure S3. Cross-tissue comparison of mean levels of DNAm between blood and five internal-target tissues for T2D 17](#_Toc46410868)

[Table S1. Description of quality control steps applied to the methylation data in the individual studies prior to the EWAS 18](#_Toc46410869)

[Table S2. Comparison of baseline population characteristics by T2D status for participants in four European cohorts included in the meta-analysis of EWAS of prevalent T2D 19](#_Toc46410870)

[Table S3. Summary of top 10 CpGs with the smallest p-value identified in association with T2D in the independent EWAS conducted by each cohort 20](#_Toc46410871)

[Table S4. Comparison of regression estimates between the fixed-effect and the random-effect meta-analysis of EWAS of prevalent T2D conducted among four European cohorts. 22](#_Toc46410872)

[Table S5. Differentially methylated regions identified in association with T2D in *comb-p* using summary estimates from the meta-analysis 24](#_Toc46410873)

[Table S6. Association between DNAm at six CpG sites identified in the meta-EWAS, and clinical phenotypes relevant to T2D among diabetes-free participants 27](#_Toc46410874)

[Table S7. Association between quartiles of DNAm at cg19693031 (*TXNIP*), and different sociodemographic, anthropometric and metabolic factors of relevance in T2D 29](#_Toc46410875)

[Table S8. Association between quartiles of DNAm at cg00144180 (*HDAC4*), and different sociodemographic, anthropometric and metabolic factors of relevance in T2D 30](#_Toc46410876)

[Table S9. Association between quartiles of DNAm at cg00574958 (*CPT1A*), and different sociodemographic, anthropometric and metabolic factors of relevance in T2D 31](#_Toc46410877)

[Table S10. Association between quartiles of DNAm at cg16765088 (near *SYNM*), and different sociodemographic, anthropometric and metabolic factors of relevance in T2D 32](#_Toc46410878)

[Table S11. Association between quartiles of DNAm at cg24704287 (near *MIR23A*), and different sociodemographic, anthropometric and metabolic factors of relevance in T2D 33](#_Toc46410879)

[Table S12. Association between quartiles of DNAm at cg06500161 (*ABCG1*), and different sociodemographic, anthropometric and metabolic factors of relevance in T2D 34](#_Toc46410880)

[Table S13. Enrichment analysis for biological processes associated with six CpG sites identified in the meta-analysis, and 77 index CpGs identified within DMRs 35](#_Toc46410881)

[Table S14. Comparison of mean values of DNA methylation between blood and five metabolically relevant tissues for T2D at six CpGs identified in the meta-analysis of EWAS 36](#_Toc46410882)

[Table S15. List of eQTMs identified in BIOS QTL in association with three of our six T2D-associated CpGs from the meta-analysis. 36](#_Toc46410883)

[Table S16. List of meQTL identified in Genetics of DNAm Consortium (GoDMC) in association with five of our six T2D-associated CpGs from the meta-analysis 36](#_Toc46410884)

[Table S17. Overlap between meQTL associated with differentially methylated CpGs in the meta-EWAS, and GWAS SNPs for different glycemic traits 37](#_Toc46410885)

## Cohort Description

### The Avon Longitudinal Study of Parents and Children (ALSPAC)

ALSPAC is a longitudinal birth cohort from the southwest of England that recruited at baseline 14,541 pregnant women with expected delivery date between 1991 and 1992 [1, 2]. Multiple behavioral, psychological, biological, genetic and epigenetic variables have been collected throughout the life course from parents and children via comprehensive participant questionnaire and visit clinics [1, 2]. Prevalent T2D was defined based on medical diagnosis, self-reported diabetes, fasting glucose ≥ 7.0 mmol/L, and/or use of medication to treat diabetes in participants aged 34-89 years from the Focus on Mothers-1 (FOM1) and Focus on Fathers-1 (FOF1) clinics, conducted ~ 20 years post-partum. Fasting blood glucose was measured using a hexokinase enzymatic method (Gluco-quant; Roche Diagnostics) at the Biochemistry department of the Glasgow Royal Infirmary, or at ALSPAC clinic laboratory facilities. For DNAm analyses, peripheral blood samples were collected 15-17 years post-partum in the adults, and DNAm was measured from purified DNA in 1,018 mother-child pairs and in 588 fathers as part of the Accessible Resource for Integrated Epigenomic Studies (ARIES) sub-study[3]. For the meta-analysis, we considered a subsample of 1,050 adults in ALSPAC with availability of variables of interest and peripheral blood DNAm. Please note that the study website contains details of all the data that is available through a fully searchable data dictionary and variable search tool: <http://www.bristol.ac.uk/alspac/researchers/our-data/>.

### The Lothian Birth Cohort of 1936 (LBC1936)

LBC1936 is a longitudinal study based in the Edinburgh area (Lothian) of Scotland. The LBC1936 study was established to understand individual differences in cognitive and brain aging and other aging outcomes of interest[4-6]. Most of the participants had taken part in the Scottish Mental Survey 1947, when an intelligence test was administered at age 11 years. They were recruited for the LBC1936 study at about age 70 years, between 2004 and 2007. In this cohort, T2D cases were defined based on levels of the hemoglobin A1c (HbA1c) ≥ 6.5% in participants aged ~ 70 years. HbA1c concentrations were measured from whole-blood samples using the Menarini HA-8160 HBA1C analyzer[7]. For DNAm analyses, peripheral blood samples were collected at approximately 70 years post-recruitment in a subset of 1,005 samples from the first wave of the main cohort[8]. DNA was extracted using standard procedures[8]. For the meta-analysis, we considered a subsample of 915 participants with complete measures of DNAm and other variables of interest.

### The Rotterdam Study (RS)

The Rotterdam Study is a large prospective population-based study in Rotterdam, the Netherlands, established to understand the determinants, incidence and progression of chronic diseases in the elderly [9, 10]. In this cohort, cases of T2D were defined based on physician diagnosis, fasting glucose ≥ 7.0 mmol/L, and/or use of anti-diabetic drugs (self-reported or from pharmacy records) in participants aged 47-87 years from the first visit of the third Rotterdam cohort (RSIII-1), and in participants aged 51-80 years from RS-Bios. The RS-Bios sub-cohort consist of participants from the third visit of the second Rotterdam cohort (RSII-3), and those from the second visit of the third cohort (RSIII-2), non-overlapping with participants from RSIII-1. In total, non-fasting glucose levels were reported in 25 (1.7%) disease-free participants from the two sub-cohorts. Fasting blood glucose was analyzed using the glucose hexokinase method within 1 week of blood sampling[11]. Blood was extracted at the Rotterdam study research facility and glucose was measured at the clinical chemistry laboratory of Erasmus University Medical Center[11]. For DNAm analyses, peripheral blood samples were collected at the first visit in RSIII-1 participants, and for RS-Bios participants blood samples were collected at the second visit in an independent sample of the RSIII cohort, and at the third visit in a subsample of the RSII cohort. DNA was extracted using standard procedures. From the core sample, we included in the meta-analysis 728 participants from RSIII-1, and 735 participants from the RS-Bios.

## Phenotypic measurement

All cohorts provided regressions adjusted for age, sex, BMI and smoking status (the preferred categorization was into three groups: never, former or current smoker). BMI was calculated as weight/height^2^. Smoking status was self-reported in most of the studies with relatively low missing rate. However, in ALSPAC, we had 23.5% missingness in self-reported smoking. To avoid excluding these samples from the EWAS, we generated a methylation score with 187 CpG sites to predict missing values for smoking status. This score had 75.9% sensitivity to identify smokers, and 90.4% specificity to discriminate non-smokers amongst ALSPAC participants with non-missing smoking data. Imputed values for smoking were categorized into never or current smokers (smoker if mean score ≥ 3.52). Detail of the method used to calculate the methylation score for smoking has been previously described [12, 13]. To control for differences in DNAm arising from cellular heterogeneity, cell proportions for six leucocyte subtypes were calculated from DNAm data [14]. In RS-Bios, direct counts for lymphocytes, monocytes and granulocytes were available for all participants.

## Methylation measurement and quality control

Each cohort used the 450K array for DNA methylation quantification. After manual inspection of raw probe intensities and sample quality, methods used for normalization of methylation values included functional normalization (ALSPAC) using the *meffil* R package [15], DASEN or SWAN (RSIII-1 & RS-Bios) options of the *WateRmelon* [16] and *minfi* [17] R packages, respectively, and using internal control probes (LBC1936) with β-values generated in the *minfi* R package [17]. Pre-processing of methylation data before the EWAS involved removal of probes that failed detection p-threshold (if p ≥ 0.01) in at least 5% of the samples, probes with detection rate < 95%, and probes located in sex chromosomes (Table S1). Cross-reactive probes, and probes prone to give spurious results that were published by Naeem *et al*.[18], were also excluded from further analyses. Normalized and pre-processed DNAm data was then used to generate surrogate variables (SVs) for batch effect adjustment, and to conduct the EWAS using multivariable linear regression models with DNAm in the β-value scale as the outcome, and T2D as the exposure.

## Quality control of individual EWAS results before meta-analysis

The *QCEWAS* R package[19] was implemented to verify summary statistics of the individual EWAS of prevalent T2D conducted by each cohort. Results were inspected to assess consistency in the distribution of effect estimates across cohorts, with upper and lower limits based on pre-specified units of measurement of the outcome (change in beta-values between 0 and 1), to identify and exclude non-informative probes with missing values for regression estimates of the EWAS, and probes located in sex chromosomes. In addition, cohorts were compared based on their precision to report effect estimates, which was proportional to the sample-size used by each study. Pruned datasets containing CpGs that surpassed QC, were then used to conduct the meta-analysis. Expected results from the inspection plots generated using *QCEWAS* were:

- Right skewed distribution of the standard error, with most of the values close to zero.
- Effect estimates centered around zero, with upper and lower limits corresponding to units of measurement of DNAm β-values.
- Correlation of expected versus observed p-values equal or close to 1.0 to rule-out data mix-up.
- Lambda (λ) or genomic inflation close to 1.0 to rule-out population stratification or non-random allocation of p-values in the sample.
- Similar distribution of effect estimates across studies, with larger studies showing a narrower distribution of effect estimates due to a better control of outliers.
- Larger studies showing better precision in estimating the effect estimate, identifying as outliers studies with large variance in the precision plot.

## Post-hoc analyses implemented in ALSPAC

We conducted multivariable regressions adjusted for age and sex between DNAm at discovered CpGs in the meta-analysis as the exposure, and various clinical phenotypes as the outcome using ALSPAC data. Phenotypes studied were glycemic traits (fasting glucose, fasting insulin, 2-h glucose and the homeostasis model assessment scores or HOMA scores), categories of glucose tolerance (controls vs prediabetes vs diabetes), anthropometric (BMI, waist-circumference) and metabolic factors (lipid markers and C-reactive protein or CRP), cardiovascular measures (systolic and diastolic blood pressure or SBP and DBP) and six white cell types (CD4^+^T, CD8^+^T, Monocytes, Granulocytes, Natural Killer cells & B cells). Variables with non-parametric distribution (HOMA scores, fasting insulin and fasting glucose, 2-h glucose, triglycerides, CRP and DBP) were log-transformed before analyses. In addition, we used DNAm at the identified CpGs stratified by quartiles to assess robustness of the association with the traits across different levels of the distribution of DNAm in the sample. P-values were determined from linear regressions for continuous variables, and from chi-squared or ordinal chi-squared tests for binary and categorical ordinal variables, respectively. We interpreted results of the analysis using continuous DNAm as a unit change or odds of the trait, per 10% increase in DNAm at the identified CpGs. For the stratified analysis, we interpreted results as a unit change or odds of the trait when comparing the lower (Q1, lower methylation) versus the top quartile of methylation (Q4, higher methylation).

To estimate the proportion of variance in T2D explained by Bonferroni significant CpG sites from the meta-analysis, and for the single DMR with the smallest corrected p-value, we used a regression model including (as predictors) all T2D-associated CpG sites, or the average methylation value across CpGs within the top DMR. Associations were adjusted for age, sex, smoking status, cell proportions and SVs in model 1, and additionally for BMI in adjusted model 2. We considered associations with T2D at p<0.05.

## Enrichment analysis for regulatory elements and look-up in publicly available databases for molecular markers associated with differentially methylated CpGs from the meta-analysis

### Enrichment analysis for regulatory elements

A *Locus Overlap Analysis* for Enrichment of Genomic Regions or LOLA [20] was used to identify regulatory elements overlapping with six T2D-associated CpG sites (*p*<1.33x10^-7^) identified in the meta-analysis, and with index CpG sites (lowest meta-analysis p-value) within Sidak-significant DMRs detected by *comb-p*[21]. For this analysis, we distinguished between hypo- and hypermethylated sets to account for potential distinct biological mechanisms. As the background region in LOLA, we selected all CpG sites included in the meta-analysis (n=368,208 autosomal CpG sites), while to test for enrichment, we used the latest LOLA core database (<http://big.databio.org/regiondb/>). Evidence of enrichment was defined as an overlap between reference and user sets with support value ≥ 5 and q-value <0.05 from the Fisher’s exact test.

### eQTMs

In an additional analysis, we searched for expression quantitative trait methylation sites or eQTMs associated with our differentially methylated CpGs from meta-analysis using the BIOS QTL browser[22]. In this repository, estimates for 12,809 unique CpG sites that correlated with 3,842 unique genes in *cis* is available at FDR<0.05 [22]. Genome-wide eQTMs were previously identified using peripheral blood DNAm and gene expression from 2,101 participants in five Dutch biobanks[22].

### meQTL

We retrieved methylation quantitative trait loci (meQTL) associated with our six differentially methylated CpGs from meta-analysis using data from the Genetics of DNAm consortium or GoDMC [23]. GoDMC is currently the largest consortium for the study of the genetics of DNAm variation. GoDMC provides association estimates for approximately 120,000,000 SNP-CpG pairs (85% *cis*-meQTL) obtained from the meta-analysis of individual meQTL analyses conducted by the collaborating studies. Sample size for meQTL detection in GoDMC is approximately 30,000 samples, largely of Caucasian origin, with mean age of 56 years. meQTL were identified using peripheral blood DNAm and retrieved at *p* <10^-8^ for *cis-*meQTL and at *p* <10^-14^ for *trans*-meQTL.

### Overlap between meQTL of interest and eQTL in specific tissues

For CpG sites of interest with an associated meQTL in blood, we investigated if the meQTL overlapped with an expression quantitative trait loci or eQTL reported in specific tissues in the GTEx dataset (GTEx_Analysis_v7)[24]. GTEx provides information of the genome-wide correlation between the genotype and tissue-specific gene expression levels. The current GTEx dataset (release V7) includes 11,688 samples and 53 tissues across 714 donors [25]. Tissues investigated in GTEx for their overlap with meQTL of interest were peripheral blood, skeletal muscle, liver, omentum or visceral fat, subcutaneous fat, pancreatic and thyroid tissue. meQTL were considered associated with gene expression at eQTL Q-value <0.05.

### Overlap between meQTL of interest and GWAS SNPs for T2D and glycemic traits

Finally, we investigated potential overlap between meQTL associated with differentially methylated CpGs from the meta-analysis, and GWAS SNPs for T2D and different glycemic traits. GWAS SNPs for T2D were retrieved from a large transethnic GWAS meta-analysis of T2D conducted by Mahajan *et al.* [26]. GWAS SNPs for the glycemic traits were retrieved from different studies with data reported in the MAGIC consortium (https://www.magicinvestigators.org/). Glycemic traits investigated included fasting insulin and fasting glucose [27], 2-hour glucose [28], hemoglobin A1c (HbA1c) [29], and the homeostasis model assessments for insulin resistance (HOMA-IR) and β-cell function (HOMA-B) [30]. We considered an meQTL to be nominally associated with T2D or the glycemic traits at GWAS p<0.05 (uncorrected p-value). Overlapping meQTL and GWAS SNPs were compared for their similarity in the effect allele and direction of effect.

## References

1. Boyd A, Golding J, Macleod J, Lawlor DA, Fraser A, Henderson J, Molloy L, Ness A, Ring S, Davey Smith G: Cohort Profile: The ‘Children of the 90s’—the index offspring of the Avon Longitudinal Study of Parents and Children. International Journal of Epidemiology 2013, 42:111-127.

2. Fraser A, Macdonald-Wallis C, Tilling K, Boyd A, Golding J, Davey Smith G, Henderson J, Macleod J, Molloy L, Ness A, et al: Cohort Profile: the Avon Longitudinal Study of Parents and Children: ALSPAC mothers cohort. Int J Epidemiol 2013, 42:97-110.

3. Relton CL, Gaunt T, McArdle W, Ho K, Duggirala A, Shihab H, Woodward G, Lyttleton O, Evans DM, Reik W, et al: Data Resource Profile: Accessible Resource for Integrated Epigenomic Studies (ARIES). International Journal of Epidemiology 2015, 44:1181-1190.

4. Deary IJ, Gow AJ, Pattie A, Starr JM: Cohort Profile: The Lothian Birth Cohorts of 1921 and 1936. International Journal of Epidemiology 2012, 41:1576-1584.

5. Deary IJ, Gow AJ, Taylor MD, Corley J, Brett C, Wilson V, Campbell H, Whalley LJ, Visscher PM, Porteous DJ, Starr JM: The Lothian Birth Cohort 1936: a study to examine influences on cognitive ageing from age 11 to age 70 and beyond. BMC Geriatrics 2007, 7:28.

6. Taylor AM, Pattie A, Deary IJ: Cohort Profile Update: The Lothian Birth Cohorts of 1921 and 1936. International Journal of Epidemiology 2018, 47:1042-1042r.

7. Altschul DM, Starr JM, Deary IJ: Cognitive function in early and later life is associated with blood glucose in older individuals: analysis of the Lothian Birth Cohort of 1936. Diabetologia 2018, 61:1946-1955.

8. Marioni RE, Shah S, McRae AF, Chen BH, Colicino E, Harris SE, Gibson J, Henders AK, Redmond P, Cox SR, et al: DNA methylation age of blood predicts all-cause mortality in later life. Genome Biology 2015, 16:25.

9. Ikram MA, Brusselle GGO, Murad SD, van Duijn CM, Franco OH, Goedegebure A, Klaver CCW, Nijsten TEC, Peeters RP, Stricker BH, et al: The Rotterdam Study: 2018 update on objectives, design and main results. Eur J Epidemiol 2017, 32:807-850.

10. Ikram MA, Brusselle G, Ghanbari M, Goedegebure A, Ikram MK, Kavousi M, Kieboom BCT, Klaver CCW, de Knegt RJ, Luik AI, et al: Objectives, design and main findings until 2020 from the Rotterdam Study. European Journal of Epidemiology 2020.

11. van der Schaft N, Schoufour JD, Nano J, Kiefte-de Jong JC, Muka T, Sijbrands EJG, Ikram MA, Franco OH, Voortman T: Dietary antioxidant capacity and risk of type 2 diabetes mellitus, prediabetes and insulin resistance: the Rotterdam Study. European journal of epidemiology 2019, 34:853-861.

12. Zeilinger S, Kuhnel B, Klopp N, Baurecht H, Kleinschmidt A, Gieger C, Weidinger S, Lattka E, Adamski J, Peters A, et al: Tobacco smoking leads to extensive genome-wide changes in DNA methylation. PLoS One 2013, 8:e63812.

13. Elliott HR, Tillin T, McArdle WL, Ho K, Duggirala A, Frayling TM, Davey Smith G, Hughes AD, Chaturvedi N, Relton CL: Differences in smoking associated DNA methylation patterns in South Asians and Europeans. Clinical Epigenetics 2014, 6:4.

14. Houseman EA, Accomando WP, Koestler DC, Christensen BC, Marsit CJ, Nelson HH, Wiencke JK, Kelsey KT: DNA methylation arrays as surrogate measures of cell mixture distribution. BMC Bioinformatics 2012, 13:86.

15. Min JL, Hemani G, Davey Smith G, Relton C, Suderman M: Meffil: efficient normalization and analysis of very large DNA methylation datasets. Bioinformatics 2018.

16. Pidsley R, Y Wong CC, Volta M, Lunnon K, Mill J, Schalkwyk LC: A data-driven approach to preprocessing Illumina 450K methylation array data. BMC Genomics 2013, 14:293.

17. Aryee MJ, Jaffe AE, Corrada-Bravo H, Ladd-Acosta C, Feinberg AP, Hansen KD, Irizarry RA: Minfi: a flexible and comprehensive Bioconductor package for the analysis of Infinium DNA methylation microarrays. Bioinformatics 2014, 30:1363-1369.

18. Naeem H, Wong NC, Chatterton Z, Hong MKH, Pedersen JS, Corcoran NM, Hovens CM, Macintyre G: Reducing the risk of false discovery enabling identification of biologically significant genome-wide methylation status using the HumanMethylation450 array. BMC Genomics 2014, 15:1-15.

19. QCEWAS: automated quality control of results of epigenome-wide association studies [<https://CRAN.R-project.org/package=QCEWAS>]

20. Sheffield NC, Bock C: LOLA: enrichment analysis for genomic region sets and regulatory elements in R and Bioconductor. Bioinformatics 2016, 32:587-589.

21. Pedersen BS, Schwartz DA, Yang IV, Kechris KJ: Comb-p: software for combining, analyzing, grouping and correcting spatially correlated P-values. Bioinformatics 2012, 28:2986-2988.

22. Bonder MJ, Luijk R, Zhernakova DV, Moed M, Deelen P, Vermaat M, van Iterson M, van Dijk F, van Galen M, Bot J, et al: Disease variants alter transcription factor levels and methylation of their binding sites. Nature Genetics 2016, 49:131.

23. Min JL, Hemani G, Hannon E, Dekkers KF, Castillo-Fernandez J, Luijk R, Carnero-Montoro E, Lawson DJ, Burrows K, Suderman M, et al: Genomic and phenomic insights from an atlas of genetic effects on DNA methylation. medRxiv 2020:2020.2009.2001.20180406.

24. The Genotype-Tissue Expression (GTEx) project [<https://www.gtexportal.org>]

25. Neumeyer S, Hemani G, Zeggini E: Strengthening Causal Inference for Complex Disease Using Molecular Quantitative Trait Loci. Trends in Molecular Medicine 2020, 26:232-241.

26. Mahajan A, Go MJ, Zhang W, Below JE, Gaulton KJ, Ferreira T, Horikoshi M, Johnson AD, Ng MC, Prokopenko I, et al: Genome-wide trans-ancestry meta-analysis provides insight into the genetic architecture of type 2 diabetes susceptibility. Nat Genet 2014, 46:234-244.

27. Fasting glucose and Fasting insulin sex-specific and sex-differentiated GWAS meta-analysis public data release May 2018 [<ftp://ftp.sanger.ac.uk/pub/magic/MAGIC-sex_dimorphic_fasting_glucose_insulin_README.pdf>]

28. Saxena R, Hivert M-F, Langenberg C, Tanaka T, Pankow JS, Vollenweider P, Lyssenko V, Bouatia-Naji N, Dupuis J, Jackson AU, et al: Genetic variation in GIPR influences the glucose and insulin responses to an oral glucose challenge. Nature Genetics 2010, 42:142-148.

29. Soranzo N, Sanna S, Wheeler E, Gieger C, Radke D, Dupuis J, Bouatia-Naji N, Langenberg C, Prokopenko I, Stolerman E, et al: Common variants at 10 genomic loci influence hemoglobin A₁(C) levels via glycemic and nonglycemic pathways. Diabetes 2010, 59:3229-3239.

30. Dupuis J, Langenberg C, Prokopenko I, Saxena R, Soranzo N, Jackson AU, Wheeler E, Glazer NL, Bouatia-Naji N, Gloyn AL, et al: New genetic loci implicated in fasting glucose homeostasis and their impact on type 2 diabetes risk. Nature genetics 2010, 42:105-116.

31. Slieker RC, Bos SD, Goeman JJ, Bovée JVMG, Talens RP, van der Breggen R, Suchiman HED, Lameijer E-W, Putter H, van den Akker EB, et al: Identification and systematic annotation of tissue-specific differentially methylated regions using the Illumina 450k array. Epigenetics & Chromatin 2013, 6:26-26.

## List of Figures and Tables

## Figure S1. Distribution of DNA methylation β-values by T2D status for six CpGs identified in the meta-analysis of EWAS of prevalent T2D.

Plots generated using ALSPAC data.


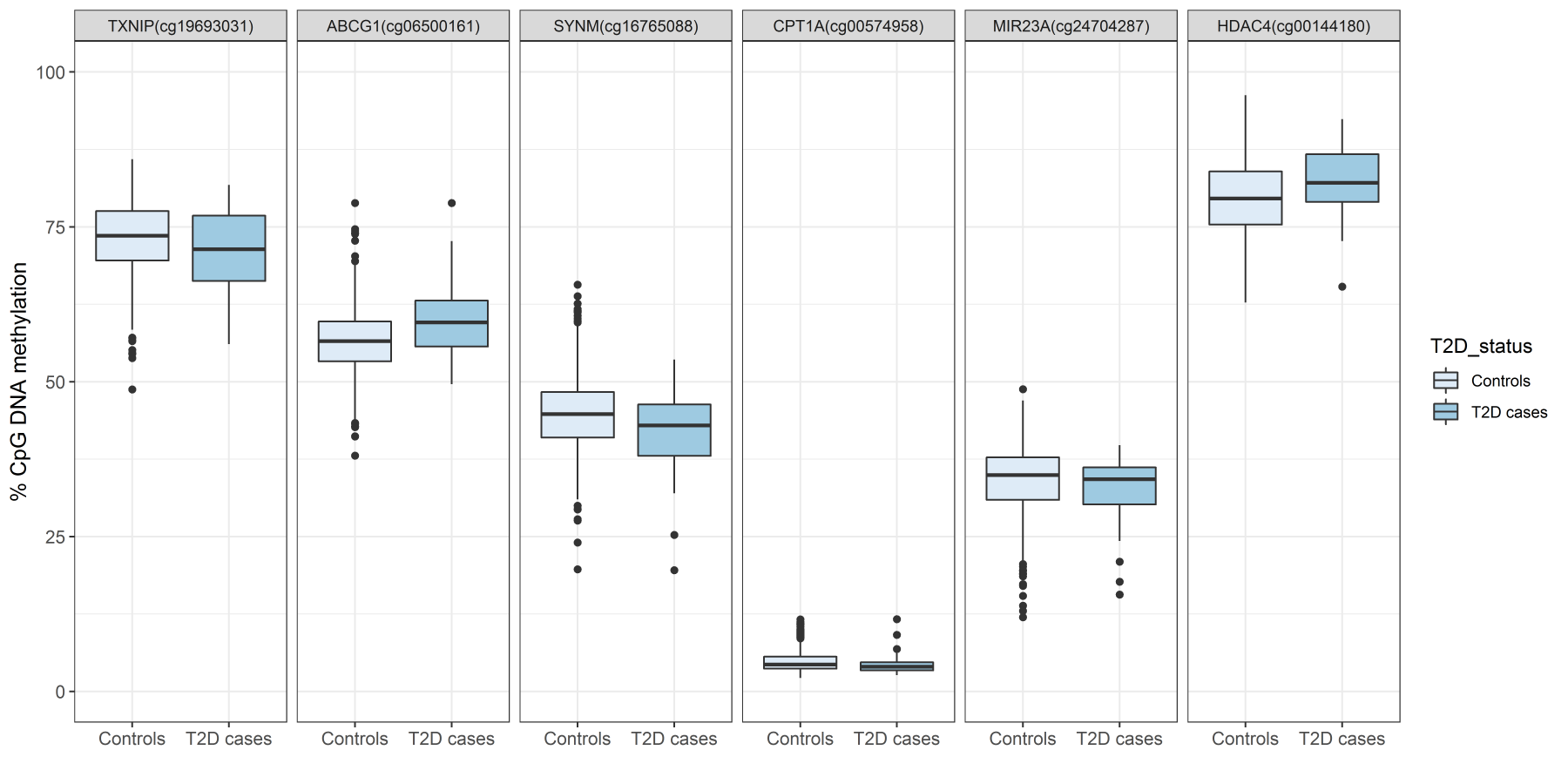


## Figure S2. Leave-one-out analysis showing the effect of removing one study at a time in results of the meta-analysis for six CpG sites identified in association with T2D.

The x-axis shows the study omitted from the analysis, and the y-axis reports the statistical significance [-log10(p-value)] of each CpG association. The horizontal black dashed line is the p-value observed in the meta-analysis; the horizontal red line is the Bonferroni corrected p-value. From left to right, studies listed in the x-axis correspond to: ALSPAC, LBC1936, RS-Bios and RSIII-1.


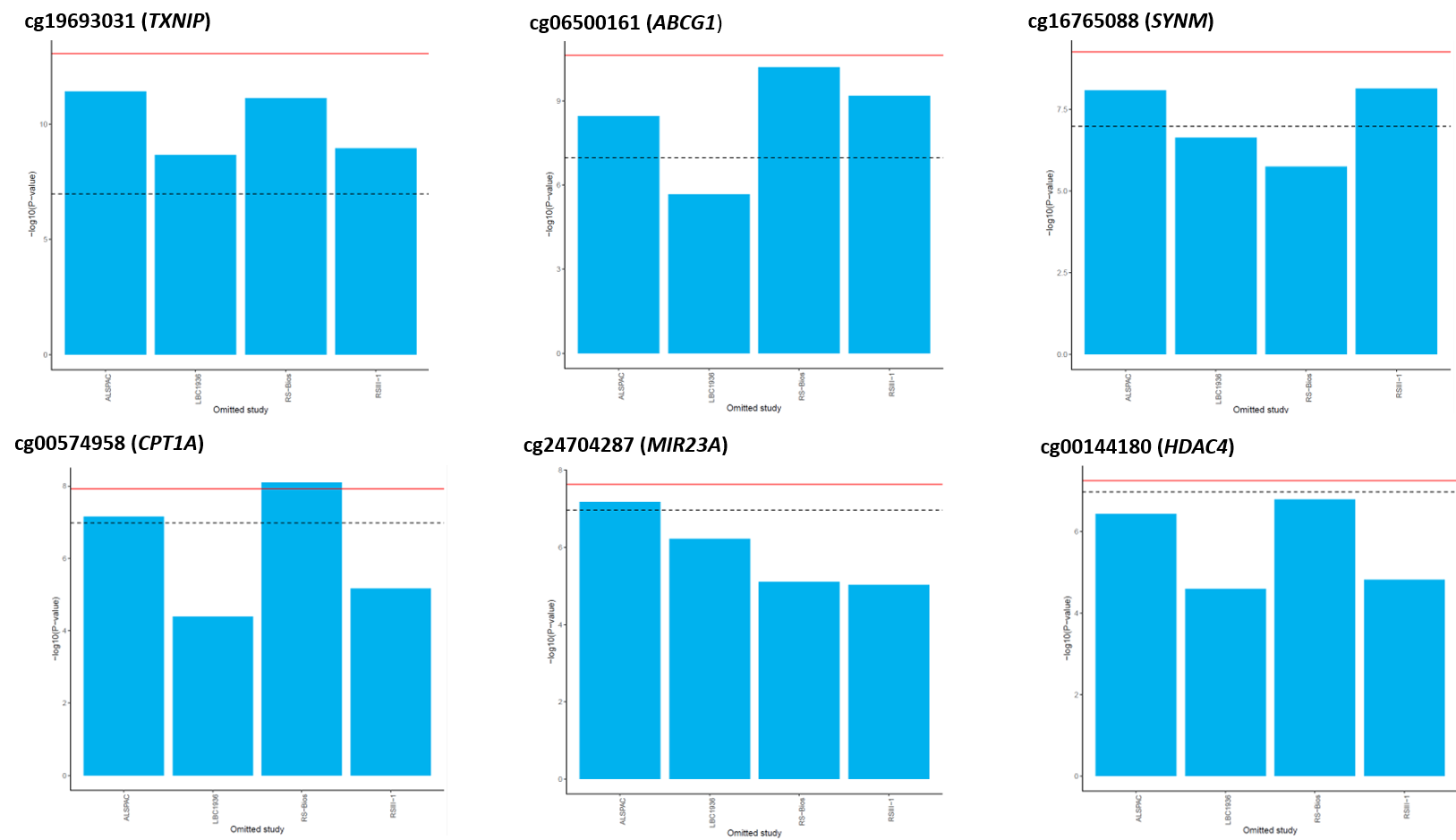


## Figure S3. Cross-tissue comparison of mean levels of DNAm between blood and five internal tissues at 80 CpG sites associated with T2D across the meta-EWAS (n= 6 sites) and DMR analyses (74 non-overlapping index CpG sites within DMRs).

Index CpGs refer to sites with the smallest meta-analysis p-value observed within each DMR. Scfat: subcutaneous fat; omentum: visceral fat. Correlation was calculated using the Pearson method. Regarding pair-wise correlation at p<0.05.


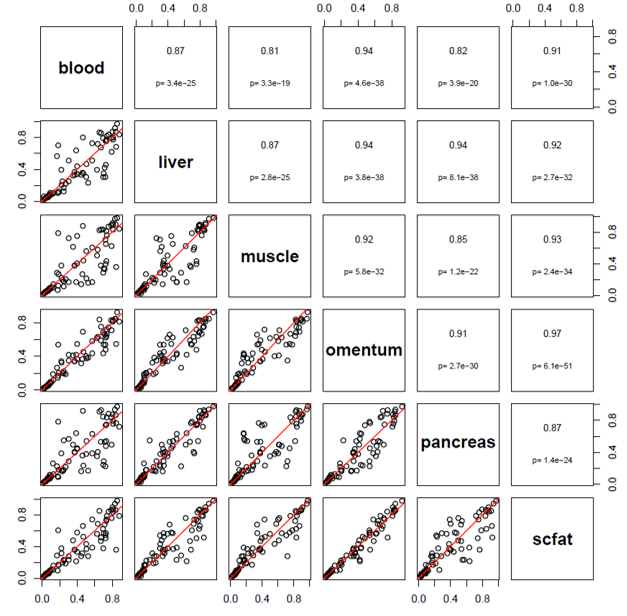


|  | ALSPAC | LBC1936 | RSIII-1 | RS-Bios |
| --- | --- | --- | --- | --- |
| **Methylation assay** | HM450 | HM450 | HM450 | HM450 |
| **Inspection of raw probe intensities** | Manual: dye staining, hybridization, nucleotide extension, bisulphite conversion | Manual: dye staining, hybridization, nucleotide extension, bisulfite conversion | Manual: specificity, hybridization, nucleotide extension, bisulfite conversion | Manual: specificity, hybridization, nucleotide extension, bisulfite conversion |
| **Normalization** | Functional Normalization, *meffil* | Control probes  *minfi* | Quantile Normalization  *lumi* | Quantile Normalization  *lumi* |
| **Detection P value cutoff** | 0.01 | 0.01 | 1e-16 | 1e-16 |
| **Sample call rate threshold** | 95% | 95% | 95% | 95% |
| **Marker call rate threshold** | 90% | 95% | 95% | 95% |
| **Sex-mismatch exclusion (samples)** | Yes | Yes | Yes | Yes |
| **Genotype-mismatch exclusion (samples)*** | Yes | Yes | No | No |
| **Number of beads filter** | 3 | 3 | 3 | 3 |
| **Total number of probes in autosomes after normalization** | 471,226 | 465,861 | 463,456 | 419,937 |
| **All covariates used in model (technical and biological)** | Age, Sex, Smoking, BMI, Houseman WBC subsets, Surrogate variables | Age, Sex, Smoking, BMI, Houseman WBC subsets, Surrogate variables | Age, Sex, Smoking, BMI, Houseman WBC subsets, Surrogate variables | Age, Sex, Smoking, BMI, direct count of WBC, Surrogate variables |

## Table S1. Description of quality control steps applied to the methylation data in the individual studies prior to the EWAS analyses.

*Based on cross-validation of genotype data for the same sample between 65 SNP-control probes in the HM450 array, and the genotyping-chip. WBC: white blood cells. Houseman WBC: predicted white blood cell counts using the Houseman algorithm based on DNAm. Counts for six cell-types were generated: CD4T, CD8T, Granulocytes, Monocytes, B-cells and Natural Killer.

## Table S2. Comparison of baseline population characteristics by T2D status for participants in four European cohorts included in the meta-analysis of EWAS of prevalent T2D.

|  | ALSPAC (N=1,050) | | | LBC1936 (N=915) | | | RSIII-1 (N=728) | | | RS-Bios (N=735) | | |
| --- | --- | --- | --- | --- | --- | --- | --- | --- | --- | --- | --- | --- |
|  | **Controls** | **Cases** | **P** | **Controls** | **Cases** | **P** | **Controls** | **Cases** | **P** | **Controls** | **Cases** | **P** |
|  | **(n=1,002)** | **(n=48)** |  | **(n=805)** | **(n=110)** |  | **(n=654)** | **(n=74)** |  | **(n=627)** | **(n=108)** |  |
| Age (years) | 49.97 | 51.94 | 1.70E-01 | 69.6 | 69.7 | 9.00E-02 | 59.55 | 63.03 | 9.00E-04 | 67.51 | 68.79 | 1.14E-01 |
| Ethnicity (n [% white]) | 948 [95] | 45 [94] | 2.50E-01 | 805 [100] | 110 [100] | --- | 654 [100] | 74 [100] | --- | 627 [100] | 108 [100] | --- |
| Fasting Glucose (mmol/L) | 5.27 | 8.45 | 2.20E-16 | 5.72† | 7.37 | 2.00E-16 | 5.33 | 8.01 | 2.00E-16 | 5.4 | 7.39 | 2.00E-16 |
| Body mass index (kg/m^2^) | 26.44 | 30.09 | 2.14E-04 | 27.4 | 30.8 | 1.20E-09 | 27.19 | 30.59 | 9.54E-07 | 27.42 | 29.35 | 1.14E-05 |
| Systolic BP (mmHg) | 122.8 | 129.45 | 8.75E-03 | 148.7 | 146.7 | 2.80E-01 | 133.8 | 140.4 | 6.23E-03 | 144.3 | 148.1 | 2.32E-02 |
| Diastolic BP (mmHg) | 73.95 | 76.82 | 4.00E-02 | 83.5 | 80.3 | 1.20E-03 | 82.92 | 82.03 | 4.83E-01 | 84.36 | 84.32 | 7.02E-01 |
| Serum Total Cholesterol (mmol/L) | 4.84 | 4.28 | 3.09E-04 | 5.54 | 4.75 | 7.70E-12 | 5.61 | 5.10 | 3.45E-04 | 5.6 | 4.94 | 1.01E-08 |
| Triglycerides (mmol/L) | 1.15 | 1.62 | 3.35E-07 | 1.59 | 1.91 | 9.80E-06 | 1.44 | 1.96 | 2.01E-05 | 1.42 | 1.75 | 4.61E-05 |
| HDL cholesterol (mmol/L) | 1.42 | 1.17 | 1.22E-04 | 1.54 | 1.34 | 5.10E-07 | 1.42 | 1.21 | 1.45E-05 | 1.55 | 1.32 | 1.31E-07 |
| LDL cholesterol (mmol/L) | 3.06 | 2.57 | 1.04E-04 | --- | --- | --- | 3.54 | 3.01 | 2.30E-05 | 3.41 | 2.83 | 3.11E-09 |
| Sex (n [% male]) | 380 [38] | 25 [19] | 5.00E-02 | 400 [50] | 62 [56] | 1.90E-01 | 294 [45] | 41 [55] | 7.59E-02 | 257 [41] | 55 [51] | 4.62E-02 |
| Physical activity* | 586 [83] | 20 [77] | 4.00E-01 | --- | --- | --- | 58.98 | 55.69 | 3.71E-01 | 60.11 | 56.03 | 3.80E-01 |
| **Estimated white cell subset**** |  |  |  |  |  |  |  |  |  |  |  |  |
| CD4T (%) | 0.17 | 0.16 | 6.10E-01 | 0.15 | 0.14 | 9.00E-02 | 0.26 | 0.25 | 4.80E-02 | 36.62 | 34.64 | 1.43E-02 |
| CD8T (%) | 0.02 | 0.03 | 8.00E-02 | 0.04 | 0.06 | 1.00E-02 | 0.09 | 0.09 | 4.30E-01 | --- | --- | --- |
| Natural Killer Cells (%) | 0.19 | 0.19 | 8.80E-01 | 0.07 | 0.08 | 6.90E-01 | 0.14 | 0.14 | 3.36E-01 | --- | --- | --- |
| B-cells (%) | 0.09 | 0.09 | 7.00E-01 | 0.07 | 0.07 | 4.70E-01 | 0.1 | 0.09 | 1.76E-01 | --- | --- | --- |
| Monocytes (%) | 0.07 | 0.08 | 5.00E-02 | 0.07 | 0.07 | 9.80E-01 | 0.08 | 0.09 | 5.92E-01 | 7.14 | 7.16 | 5.55E-01 |
| Gran (%) | 0.51 | 0.50 | 3.30E-01 | 0.63 | 0.62 | 5.70E-01 | 0.37 | 0.40 | 1.32E-01 | 6.75 | 7.35 | 5.66E-03 |
| **Smoking (n [%])** |  |  |  |  |  |  |  |  |  |  |  |  |
| Never smoker | 912 [91] | 41 [85] | 1.90E-01 | 386 [48] | 44 [40] | 2.40E-01 | 197 [30] | 15 [20] | 8.72E-02 | 227 [36] | 27 [25] | 6.01E-02 |
| Ever smoker | --- | --- |  | 332 [41] | 50 [45] |  | 279 [43] | 41 [55] |  | 338 [54] | 66 [61] |  |
| Current smoker | 90 [9] | 7 [15] |  | 87 [11] | 16 [15] |  | 178 [27] | 18 [24] |  | 62 [10] | 15 [14] |  |

Continuous variables were described using the mean, while categorical variables using the frequency and proportion. *Physical activity was measured as a continuous variable in the Rotterdam Studies using the metabolic equivalent (MET) score in hours/week. ** Direct count for lymphocytes, granulocytes and monocytes, was available for all participants in RS-Bios. Mean value of HbA1c (%) in LBC1936. † Mean values of HbA1c (%) reported for participants in the LBC1936 cohort.

## Table S3. Summary of top 10 CpGs with the smallest p-value identified in association with T2D in the independent EWAS conducted by each cohort.

Showing regression estimates for the main model, and for the model additionally adjusted for BMI. Results in main model were adjusted for age, sex, SVs, cellular heterogeneity and smoking, and for BMI in a secondary model. Genomic inflation factor (λ) for the EWAS in ALSPAC: Main model λ=1.46, BMI-Model λ=1.44; for the EWAS in LBC1936: Main model λ=1.07, BMI-Model λ=1.08.

|  | **ALSPAC (N=1,050)** | | | | **LBC1936 (N=915)** | | | |
| --- | --- | --- | --- | --- | --- | --- | --- | --- |
|  | CpG | Loci | Beta | P | CpG | Loci | Beta | P |
| Main Model | cg14045803 | *STARD10* | -0.011 | 3.07E-07 | cg06500161 | *ABCG1* | 0.024 | 5.18E-08 |
|  | cg15986668 | *NFYC* | -0.065 | 5.78E-07 | cg09371351 | *HSD3B2* | 0.04 | 2.31E-06 |
|  | cg10870892 | *CTTN* | -0.045 | 7.58E-07 | cg07051796 | *ZFHX3* | -0.012 | 3.40E-06 |
|  | cg19823491 | *OTX1* | -0.006 | 1.70E-06 | cg19693031 | *TXNIP* | -0.026 | 3.88E-06 |
|  | cg00204249 | *DNAH17* | -0.014 | 2.47E-06 | cg27243685 | *ABCG1* | 0.016 | 4.65E-06 |
|  | cg04016326 | *GRIN2B* | -0.055 | 2.78E-06 | cg17055821 | *C17orf75* | 0.025 | 6.22E-06 |
|  | cg26652413 | *CPAMD8* | -0.022 | 3.49E-06 | cg20068400 | *Intergenic* | -0.029 | 7.41E-06 |
|  | cg03206717 | *SLC25A38* | -0.003 | 4.84E-06 | cg13565670 | *FBRSL1* | 0.01 | 9.33E-06 |
|  | cg14290451 | *RPL10A* | -0.004 | 5.20E-06 | cg03312117 | *Intergenic* | -0.041 | 9.35E-06 |
|  | cg24605023 | *CADPS* | -0.028 | 6.06E-06 | cg15127702 | *EMID2* | -0.019 | 9.62E-06 |
| BMI-adjusted model | cg14045803 | *STARD10* | -0.012 | 1.39E-07 | cg06500161 | *ABCG1* | 0.017 | 1.22E-04 |
|  | cg15986668 | *NFYC* | -0.071 | 5.48E-08 | cg09371351 | *HSD3B2* | 0.042 | 1.23E-06 |
|  | cg10870892 | *CTTN* | -0.045 | 1.13E-06 | cg07051796 | *ZFHX3* | -0.013 | 3.47E-07 |
|  | cg19823491 | *OTX1* | -0.006 | 2.99E-06 | cg19693031 | *TXNIP* | -0.025 | 1.75E-05 |
|  | cg00204249 | *DNAH17* | -0.015 | 2.76E-06 | cg27243685 | *ABCG1* | 0.013 | 2.07E-04 |
|  | cg04016326 | *GRIN2B* | -0.054 | 5.71E-06 | cg17055821 | *C17orf75* | 0.027 | 4.47E-06 |
|  | cg26652413 | *CPAMD8* | -0.023 | 2.51E-06 | cg20068400 | *Intergenic* | -0.030 | 7.02E-06 |
|  | cg03206717 | *SLC25A38* | -0.003 | 2.95E-06 | cg13565670 | *FBRSL1* | 0.011 | 1.65E-06 |
|  | cg14290451 | *RPL10A* | -0.004 | 1.35E-05 | cg03312117 | *Intergenic* | -0.040 | 2.43E-05 |
|  | cg24605023 | *CADPS* | -0.026 | 2.53E-05 | cg15127702 | *EMID2* | -0.018 | 2.93E-05 |

**Table S3.** (Continued)

Genomic inflation factor (λ) for the EWAS in RSIII-1: Main model λ=1.15, BMI-Model λ=1.07; for the EWAS in RS-Bios: Main model λ=1.04, BMI-Model λ=1.03.

|  | **RSIII-1 (N=728)** | | | | **RS-Bios (N=735)** | | | |
| --- | --- | --- | --- | --- | --- | --- | --- | --- |
|  | CpG | Loci | Beta | P | CpG | Loci | Beta | P |
| Main Model | cg14278808 | *LOC157627* | 0.020 | 2.75E-07 | cg05059607 | *PITPNC1* | 0.021 | 1.95E-06 |
|  | cg16330965 | *SNAPC5* | -0.012 | 4.79E-07 | cg24795867 | *WNT5B* | -0.008 | 2.29E-06 |
|  | cg13212575 | *MAEL* | -0.009 | 9.15E-07 | cg16339915 | *TIFAB* | -0.007 | 3.65E-06 |
|  | cg02484673 | *JPH3* | -0.012 | 1.32E-06 | cg14491707 | *CACNA1B* | -0.016 | 4.00E-06 |
|  | cg05887092 | *PGS1* | -0.012 | 3.15E-06 | cg02243386 | *XIRP1* | -0.008 | 7.44E-06 |
|  | cg08121984 | *APOC1P1* | -0.012 | 3.88E-06 | cg11983038 | *Intergenic* | -0.025 | 8.82E-06 |
|  | cg04845819 | *PRR23C* | -0.011 | 4.26E-06 | cg24512093 | *ROBO1* | -0.011 | 1.05E-05 |
|  | cg21193660 | *Intergenic* | -0.017 | 5.79E-06 | cg01316152 | *Intergenic* | -0.008 | 1.37E-05 |
|  | cg05084700 | *Intergenic* | -0.011 | 6.18E-06 | cg10500218 | *IER5* | 0.006 | 1.54E-05 |
|  | cg00574958 | *CPT1A* | -0.018 | 6.19E-06 | cg18143317 | *Intergenic* | -0.007 | 1.64E-05 |
| BMI-adjusted model | cg14278808 | *LOC157627* | 0.020 | 1.04E-06 | cg05059607 | *PITPNC1* | 0.019 | 1.89E-05 |
|  | cg16330965 | *SNAPC5* | -0.013 | 5.00E-07 | cg24795867 | *WNT5B* | -0.008 | 6.61E-06 |
|  | cg13212575 | *MAEL* | -0.008 | 9.47E-06 | cg16339915 | *TIFAB* | -0.007 | 3.32E-06 |
|  | cg02484673 | *JPH3* | -0.012 | 2.01E-06 | cg14491707 | *CACNA1B* | -0.015 | 1.05E-05 |
|  | cg05887092 | *PGS1* | -0.013 | 3.20E-06 | cg02243386 | *XIRP1* | -0.008 | 4.41E-05 |
|  | cg08121984 | *APOC1P1* | -0.012 | 6.82E-06 | cg11983038 | *Intergenic* | -0.025 | 9.82E-06 |
|  | cg04845819 | *PRR23C* | -0.010 | 4.92E-05 | cg24512093 | *ROBO1* | -0.011 | 1.59E-05 |
|  | cg21193660 | *Intergenic* | -0.015 | 8.38E-05 | cg01316152 | *Intergenic* | -0.008 | 3.03E-05 |
|  | cg05084700 | *Intergenic* | -0.010 | 1.98E-05 | cg10500218 | *IER5* | 0.006 | 2.49E-05 |
|  | cg00574958 | *CPT1A* | -0.013 | 1.10E-03 | cg18143317 | *Intergenic* | -0.007 | 4.68E-05 |
|  | | | | | | | | |

## Table S4. Comparison of regression estimates between the fixed-effect and the random-effect meta-analysis of EWAS of prevalent T2D conducted among four European cohorts.

CpG sites compared were identified in association with T2D at *p* <1.0x10^-5^ in the fixed-effect analysis, adjusting for age, sex, SVs, cellular heterogeneity and smoking. Direction corresponds to the relative effect detected at the individual CpG site in the EWAS in ALSPAC, LBC1936, RSIII-1 and RS-Bios, respectively. Highlighted in bold are associations identified with Bonferroni significance at p <0.05 in the fixed-effect and random-effect model.

| CpG site | Chr | Gene | Direction | **Fixed-Effect IVW Meta-analysis** | | | | | **Random-Effect Meta-analysis** | | | |
| --- | --- | --- | --- | --- | --- | --- | --- | --- | --- | --- | --- | --- |
|  |  |  |  | Beta | P | Bonferroni | *I^2^* | P_het_ | Beta | P | Tau | Bonferroni |
| **cg19693031** | 1 | ***TXNIP*** | ---- | -0.019 | 8.75E-14 | **3.29E-08** | 0.00 | 0.455 | -0.019 | 8.75E-14 | 0.00E+00 | **3.29E-08** |
| cg06500161 | 21 | *ABCG1* | ++++ | 0.013 | 2.34E-11 | 8.81E-06 | 77.70 | 0.004 | 0.015 | 3.62E-04 | 5.61E-05 | 1.00E+00 |
| **cg16765088** | 15 | ***SYNM*** | ---- | -0.011 | 5.50E-10 | **2.07E-04** | 0.00 | 0.429 | -0.011 | 5.50E-10 | 0.00E+00 | **2.07E-04** |
| cg00574958 | 11 | *CPT1A* | ---- | -0.007 | 1.20E-08 | 4.53E-03 | 72.20 | 0.013 | -0.008 | 1.25E-03 | 1.71E-05 | 1.00E+00 |
| **cg24704287** | 19 | ***MIR23A*** | ---- | -0.011 | 2.34E-08 | **8.79E-03** | 0.00 | 0.968 | -0.011 | 2.34E-08 | 0.00E+00 | **8.79E-03** |
| **cg00144180** | 2 | ***HDAC4*** | ++++ | 0.012 | 5.64E-08 | **2.12E-02** | 4.70 | 0.369 | 0.012 | 1.21E-07 | 1.00E-06 | **4.56E-02** |
| cg04567334 | 10 | *CDH23* | ---- | -0.006 | 1.67E-07 | 6.30E-02 | 0.00 | 0.670 | -0.006 | 1.67E-07 | 0.00E+00 | 6.30E-02 |
| cg10584271 | 3 | *ITIH1* | ---- | -0.014 | 1.73E-07 | 6.52E-02 | 5.60 | 0.365 | -0.014 | 4.28E-07 | 1.70E-06 | 1.61E-01 |
| cg26270261 | 12 | *KRT4* | ---- | -0.006 | 5.68E-07 | 2.14E-01 | 31.10 | 0.225 | -0.007 | 5.95E-05 | 3.40E-06 | 1.00E+00 |
| cg16575444 | 16 | *CX3CL1* | +--- | -0.006 | 6.83E-07 | 2.57E-01 | 0.00 | 0.454 | -0.006 | 6.83E-07 | 0.00E+00 | 2.57E-01 |
| cg24512093 | 3 | *ROBO1* | ---- | -0.010 | 7.16E-07 | 2.69E-01 | 16.50 | 0.309 | -0.009 | 3.13E-05 | 3.70E-06 | 1.00E+00 |
| cg11983038 | 13 | *Intergenic* | ---- | -0.017 | 7.23E-07 | 2.72E-01 | 65.10 | 0.035 | -0.016 | 7.18E-03 | 8.99E-05 | 1.00E+00 |
| cg25136644 | 7 | *ATG9B* | ---- | -0.007 | 7.27E-07 | 2.74E-01 | 34.80 | 0.204 | -0.008 | 7.52E-05 | 5.10E-06 | 1.00E+00 |
| cg20812370 | 1 | *PBX1* | ---- | -0.007 | 7.40E-07 | 2.78E-01 | 70.70 | 0.017 | -0.009 | 2.65E-03 | 2.15E-05 | 1.00E+00 |
| cg24686009 | 12 | *RAP1B* | ---+ | -0.002 | 1.19E-06 | 4.46E-01 | 0.20 | 0.391 | -0.002 | 1.24E-06 | 0.00E+00 | 4.67E-01 |
| cg11024682 | 17 | *SREBF1* | ++++ | 0.008 | 1.33E-06 | 5.01E-01 | 0.00 | 0.694 | 0.008 | 1.33E-06 | 0.00E+00 | 5.01E-01 |
| cg06114363 | 1 | *ZNF683* | ---? | -0.010 | 1.37E-06 | 5.14E-01 | 0.00 | 0.530 | -0.010 | 1.37E-06 | 0.00E+00 | 5.14E-01 |
| cg01963618 | 6 | *LOC285768* | ---- | -0.008 | 1.55E-06 | 5.84E-01 | 0.00 | 0.801 | -0.008 | 1.55E-06 | 0.00E+00 | 5.84E-01 |
| cg22680424 | 11 | *HCCA2* | ++++ | 0.008 | 2.16E-06 | 8.13E-01 | 0.00 | 0.492 | 0.008 | 2.16E-06 | 0.00E+00 | 8.13E-01 |
| cg19876302 | 10 | *Intergenic* | ---- | -0.008 | 2.22E-06 | 8.36E-01 | 0.00 | 0.430 | -0.008 | 2.22E-06 | 0.00E+00 | 8.36E-01 |
| cg08857797 | 17 | *VPS25* | ++++ | 0.009 | 2.28E-06 | 8.56E-01 | 0.00 | 0.813 | 0.009 | 2.28E-06 | 0.00E+00 | 8.56E-01 |
| cg27374726 | 10 | *Intergenic* | ---- | -0.009 | 2.32E-06 | 8.72E-01 | 42.40 | 0.157 | -0.009 | 4.48E-04 | 1.03E-05 | 1.00E+00 |
| cg09185884 | 17 | *KCTD2* | ++++ | 0.011 | 2.33E-06 | 8.75E-01 | 0.00 | 0.919 | 0.011 | 2.33E-06 | 0.00E+00 | 8.75E-01 |
| cg27115863 | 22 | *Intergenic* | ---- | -0.011 | 2.41E-06 | 9.07E-01 | 13.40 | 0.325 | -0.011 | 1.52E-05 | 3.60E-06 | 1.00E+00 |
| cg24795867 | 12 | *WNT5B* | ---- | -0.006 | 2.47E-06 | 9.29E-01 | 43.20 | 0.152 | -0.005 | 1.12E-02 | 6.80E-06 | 1.00E+00 |
| cg08945443 | 10 | *ZMYND17* | -+++ | 0.010 | 2.64E-06 | 9.94E-01 | 51.70 | 0.102 | 0.009 | 9.86E-03 | 2.52E-05 | 1.00E+00 |
| cg06039489 | 20 | *C20orf26* | ++++ | 0.016 | 2.71E-06 | 1.00E+00 | 16.80 | 0.308 | 0.016 | 2.98E-05 | 9.70E-06 | 1.00E+00 |

**Table S4.** (Continued)

| CpG site | Chr | Gene | Direction | **Fixed-Effect inverse-variance weighted**  **Meta-analysis** | | | | | **Random-Effect Meta-analysis** | | | |
| --- | --- | --- | --- | --- | --- | --- | --- | --- | --- | --- | --- | --- |
|  |  |  |  | Beta | P | Bonferroni | *I^2^* | P_het_ | Beta | P | Tau | Bonferroni |
| cg08570691 | 19 | *RPL13AP5* | ---- | -0.008 | 2.78E-06 | 1.00E+00 | 42.60 | 0.156 | -0.009 | 2.70E-04 | 1.05E-05 | 1.00E+00 |
| cg12593793 | 1 | *Intergenic* | ---- | -0.008 | 2.90E-06 | 1.00E+00 | 60.50 | 0.055 | -0.009 | 1.32E-03 | 1.84E-05 | 1.00E+00 |
| cg27037013 | 21 | *Intergenic* | ---- | -0.015 | 2.90E-06 | 1.00E+00 | 51.90 | 0.101 | -0.017 | 5.58E-04 | 5.04E-05 | 1.00E+00 |
| cg07212837 | 8 | *Intergenic* | ++++ | 0.006 | 3.28E-06 | 1.00E+00 | 0.00 | 0.843 | 0.006 | 3.28E-06 | 0.00E+00 | 1.00E+00 |
| cg16192197 | 6 | *Intergenic* | ++++ | 0.010 | 3.71E-06 | 1.00E+00 | 0.00 | 0.503 | 0.010 | 3.71E-06 | 0.00E+00 | 1.00E+00 |
| cg15560632 | 7 | *LRCH4* | ---- | -0.001 | 3.83E-06 | 1.00E+00 | 3.50 | 0.375 | -0.001 | 6.95E-06 | 0.00E+00 | 1.00E+00 |
| cg14003143 | 20 | *SGK2* | ---- | -0.006 | 4.12E-06 | 1.00E+00 | 62.60 | 0.045 | -0.007 | 1.73E-03 | 1.27E-05 | 1.00E+00 |
| cg20154947 | 8 | *PLEC1* | ---- | -0.002 | 4.34E-06 | 1.00E+00 | 23.40 | 0.271 | -0.002 | 1.51E-04 | 2.00E-07 | 1.00E+00 |
| cg25741837 | 2 | *SMYD5* | ++++ | 0.009 | 4.76E-06 | 1.00E+00 | 0.00 | 0.396 | 0.009 | 4.76E-06 | 0.00E+00 | 1.00E+00 |
| cg26766064 | 17 | *MIR657* | ---- | -0.007 | 5.17E-06 | 1.00E+00 | 0.00 | 0.412 | -0.007 | 5.17E-06 | 0.00E+00 | 1.00E+00 |
| cg25536676 | 1 | *DHCR24* | ---- | -0.008 | 5.39E-06 | 1.00E+00 | 54.80 | 0.084 | -0.009 | 1.40E-03 | 1.65E-05 | 1.00E+00 |
| cg11376147 | 11 | *SLC43A1* | ---- | -0.006 | 5.43E-06 | 1.00E+00 | 0.00 | 0.764 | -0.006 | 5.43E-06 | 0.00E+00 | 1.00E+00 |
| cg20316538 | 2 | *RUFY4* | ---- | -0.005 | 6.11E-06 | 1.00E+00 | 0.00 | 0.605 | -0.005 | 6.11E-06 | 0.00E+00 | 1.00E+00 |
| cg18181703 | 17 | *SOCS3* | ---- | -0.010 | 6.20E-06 | 1.00E+00 | 56.50 | 0.075 | -0.012 | 1.46E-03 | 3.03E-05 | 1.00E+00 |
| cg11851382 | 1 | *PPAP2B* | ---- | -0.008 | 6.42E-06 | 1.00E+00 | 0.00 | 0.491 | -0.008 | 6.42E-06 | 0.00E+00 | 1.00E+00 |
| cg00162348 | 16 | *RNF40* | ---- | -0.002 | 6.64E-06 | 1.00E+00 | 0.00 | 0.600 | -0.002 | 6.64E-06 | 0.00E+00 | 1.00E+00 |
| cg07184465 | 5 | *SPZ1* | ---- | -0.007 | 7.18E-06 | 1.00E+00 | 38.20 | 0.183 | -0.007 | 3.75E-04 | 6.60E-06 | 1.00E+00 |
| cg14284506 | 17 | *Intergenic* | ---- | -0.005 | 7.31E-06 | 1.00E+00 | 21.90 | 0.279 | -0.005 | 1.76E-04 | 1.50E-06 | 1.00E+00 |
| cg11252555 | 19 | *RPL13AP5* | ---- | -0.008 | 7.44E-06 | 1.00E+00 | 63.30 | 0.043 | -0.010 | 2.54E-03 | 2.40E-05 | 1.00E+00 |
| cg10082515 | 7 | *Intergenic* | ---- | -0.013 | 7.46E-06 | 1.00E+00 | 0.00 | 0.505 | -0.013 | 7.46E-06 | 0.00E+00 | 1.00E+00 |
| cg00896068 | 13 | *Intergenic* | ---- | -0.008 | 7.58E-06 | 1.00E+00 | 0.00 | 0.482 | -0.008 | 7.58E-06 | 0.00E+00 | 1.00E+00 |
| cg01577083 | 16 | *Intergenic* | ---- | -0.011 | 7.93E-06 | 1.00E+00 | 0.00 | 0.621 | -0.011 | 7.93E-06 | 0.00E+00 | 1.00E+00 |
| cg00320980 | 10 | *Intergenic* | ---- | -0.009 | 7.97E-06 | 1.00E+00 | 0.00 | 0.744 | -0.009 | 7.97E-06 | 0.00E+00 | 1.00E+00 |
| cg20231084 | 11 | *Intergenic* | ---- | -0.006 | 8.36E-06 | 1.00E+00 | 0.00 | 0.796 | -0.006 | 8.36E-06 | 0.00E+00 | 1.00E+00 |
| cg15832662 | 11 | *RTN3* | ---- | -0.009 | 8.45E-06 | 1.00E+00 | 0.00 | 0.829 | -0.009 | 8.45E-06 | 0.00E+00 | 1.00E+00 |
| cg13178597 | 6 | *RGS17* | ---- | -0.010 | 8.57E-06 | 1.00E+00 | 0.00 | 0.580 | -0.010 | 8.57E-06 | 0.00E+00 | 1.00E+00 |
| cg20116935 | 3 | *SEMA3B* | ---- | -0.006 | 8.89E-06 | 1.00E+00 | 4.80 | 0.369 | -0.006 | 1.64E-05 | 4.00E-07 | 1.00E+00 |
| cg00989505 | 14 | *MIR299* | +--- | -0.004 | 9.33E-06 | 1.00E+00 | 14.80 | 0.318 | -0.004 | 1.05E-04 | 7.00E-07 | 1.00E+00 |
| cg07068382 | 6 | *MTCH1* | -+++ | 0.010 | 9.46E-06 | 1.00E+00 | 46.70 | 0.131 | 0.011 | 2.07E-03 | 2.17E-05 | 1.00E+00 |
| cg14476101 | 1 | *PHGDH* | ---- | -0.015 | 9.46E-06 | 1.00E+00 | 52.00 | 0.100 | -0.015 | 3.33E-03 | 5.11E-05 | 1.00E+00 |
| cg20456243 | 2 | *SPEG* | ---- | -0.007 | 9.99E-06 | 1.00E+00 | 0.00 | 0.490 | -0.007 | 9.99E-06 | 0.00E+00 | 1.00E+00 |

## Table S5. Differentially methylated regions identified in association with T2D in *comb-p* using summary estimates from the meta-analysis.

Results correspond to the model adjusted for age, sex, SVs, cells and smoking. Effect size is measured as % Meth, representing the median absolute percent change in methylation between T2D cases and controls for all the CpGs in the region. Direction represents the relative effect observed in the region across all CpG sites. Index CpG is the site within the region with the smallest p-value from the meta-analysis. P-region is the uncorrected p-value of the region, and Sidak is the p-value adjusted for multiple testing. Showing 77 regions identified with Sidak significance at p <0.05.

| Chr | DMR | Nearest  Gene | Size (bp) | CpG  count | % Meth | Direction | Index CpG | Lowest P | P_region_ | Sidak |
| --- | --- | --- | --- | --- | --- | --- | --- | --- | --- | --- |
| 1 | Chr1:108023249-108023483 | *NTNG1* | 234 | 5 | 1.07 | Hyper | cg20016673 | 5.94E-03 | 3.06E-05 | 4.80E-02 |
| 1 | Chr1:120255941-120255993 | *PHGDH* | 52 | 2 | 1.26 | Hypo | cg14476101 | 9.46E-06 | 5.01E-08 | 3.62E-04 |
| 1 | Chr1:145441552-145441553 | *TXNIP* | 1 | 1 | 1.93 | Hypo | cg19693031 | 8.75E-14 | 8.75E-14 | 3.29E-08 |
| 1 | Chr1:181074635-181074791 | *Intergenic* | 156 | 3 | 0.15 | Hypo | cg12074154 | 4.05E-04 | 7.79E-06 | 1.86E-02 |
| 1 | Chr1:19600454-19600913 | *AKR7L* | 459 | 7 | 0.87 | Hyper | cg13935437 | 2.44E-03 | 2.37E-05 | 1.92E-02 |
| 1 | Chr1:201708500-201708789 | *NAV1* | 289 | 5 | 0.71 | Hypo | cg07139329 | 1.23E-03 | 4.04E-06 | 5.25E-03 |
| 1 | Chr1:223317364-223317549 | *TLR5* | 185 | 4 | 0.64 | Hyper | cg18201671 | 6.72E-05 | 2.26E-06 | 4.59E-03 |
| 1 | Chr1:228890801-228891037 | *Intergenic* | 236 | 5 | 1.02 | Hyper | cg23012917 | 2.59E-03 | 1.19E-05 | 1.88E-02 |
| 1 | Chr1:28573736-28573941 | *Intergenic* | 205 | 4 | 0.26 | Hypo | cg07913096 | 9.88E-05 | 2.42E-07 | 4.44E-04 |
| 1 | Chr1:28906210-28906539 | *SNHG12* | 329 | 5 | 0.51 | Hypo | cg26328951 | 1.84E-05 | 8.97E-09 | 1.03E-05 |
| 1 | Chr1:36023025-36023429 | *NCDN* | 404 | 8 | 0.10 | Hypo | cg10905247 | 1.23E-04 | 7.02E-06 | 6.52E-03 |
| 1 | Chr1:44457124-44457407 | *CCDC24* | 283 | 6 | 0.10 | Hypo | cg04921669 | 1.88E-04 | 5.70E-06 | 7.54E-03 |
| 1 | Chr1:61548526-61549011 | *NFIA* | 485 | 4 | 0.10 | Hypo | cg11531787 | 3.24E-03 | 4.04E-05 | 3.09E-02 |
| 1 | Chr1:92012615-92012737 | *Intergenic* | 122 | 3 | 1.46 | Hypo | cg25838465 | 2.01E-05 | 4.03E-06 | 1.24E-02 |
| 2 | Chr2:11123476-11123617 | *Intergenic* | 141 | 3 | 0.89 | Hyper | cg02479842 | 1.22E-03 | 1.56E-06 | 4.16E-03 |
| 2 | Chr2:120124292-120124678 | *C2orf76* | 386 | 10 | 0.07 | Hyper | cg08326511 | 4.16E-03 | 4.59E-06 | 4.47E-03 |
| 2 | Chr2:231692812-231693071 | *Intergenic* | 259 | 3 | 0.76 | Hypo | cg19184455 | 3.00E-05 | 8.71E-06 | 1.26E-02 |
| 2 | Chr2:233284402-233284662 | *Intergenic* | 260 | 2 | 1.11 | Hypo | cg05951221 | 1.78E-04 | 3.53E-05 | 4.98E-02 |
| 2 | Chr2:239046879-239047337 | *KLHL30* | 458 | 5 | 0.51 | Hypo | cg14597586 | 4.35E-04 | 2.64E-08 | 2.17E-05 |
| 2 | Chr2:240294246-240294363 | *HDAC4* | 117 | 2 | 0.72 | Hyper | cg00144180 | 5.64E-08 | 2.11E-06 | 6.75E-03 |
| 2 | Chr2:32390673-32390938 | *SLC30A6* | 265 | 5 | 0.10 | Hypo | cg04270736 | 3.94E-04 | 4.64E-06 | 6.57E-03 |
| 2 | Chr2:65593761-65593934 | *SPRED2* | 173 | 3 | 0.94 | Hypo | cg01751245 | 3.15E-04 | 1.48E-06 | 3.21E-03 |
| 2 | Chr2:68592345-68592395 | *PLEK* | 50 | 4 | 0.56 | Hyper | cg04872689 | 3.12E-04 | 4.06E-06 | 3.01E-02 |
| 2 | Chr2:74669349-74669517 | *RTKN* | 168 | 5 | 0.80 | Hypo | cg26090072 | 3.44E-04 | 3.79E-06 | 8.45E-03 |

**Table S5.** (Continued)

| Chr | DMR | Nearest  Gene | Size (bp) | CpG  count | % Meth | Direction | Index CpG | Lowest P | P-region | Sidak |
| --- | --- | --- | --- | --- | --- | --- | --- | --- | --- | --- |
| 3 | Chr3:4534791-4535155 | *ITPR1* | 364 | 9 | 0.10 | Hypo | cg02808075 | 1.61E-04 | 1.17E-05 | 1.21E-02 |
| 4 | Chr4:57333365-57333860 | *SRP72* | 495 | 6 | 0.11 | Hypo | cg03129910 | 2.79E-04 | 2.73E-05 | 2.06E-02 |
| 5 | Chr5:136340060-136340208 | *SPOCK1* | 148 | 2 | 0.59 | Hypo | cg18998172 | 2.78E-04 | 9.33E-06 | 2.34E-02 |
| 5 | Chr5:139488181-139488624 | *Intergenic* | 443 | 3 | 0.59 | Hypo | cg00396158 | 4.21E-04 | 2.29E-06 | 1.94E-03 |
| 5 | Chr5:139927110-139927472 | *YIF1B* | 362 | 10 | 0.14 | Hypo | cg25419506 | 1.67E-04 | 1.53E-06 | 1.59E-03 |
| 5 | Chr5:143978290-143978421 | *Intergenic* | 131 | 4 | 1.22 | Hyper | cg02030403 | 9.17E-04 | 5.36E-07 | 1.54E-03 |
| 6 | Chr6:125283726-125283970 | *STL* | 244 | 3 | 0.41 | Hypo | cg07439975 | 5.78E-03 | 2.00E-05 | 3.04E-02 |
| 6 | Chr6:13574034-13574574 | *SIRT5* | 540 | 6 | 0.27 | Hyper | cg07534331 | 2.25E-04 | 1.41E-05 | 9.79E-03 |
| 6 | Chr6:149806635-149806733 | *ZC3H12D* | 98 | 2 | 0.93 | Hypo | cg06762457 | 6.85E-05 | 5.13E-06 | 1.95E-02 |
| 6 | Chr6:5261091-5261561 | *LYRM4* | 470 | 10 | 0.10 | Hypo | cg15638207 | 1.33E-03 | 2.08E-05 | 1.65E-02 |
| 6 | Chr6:72130742-72131021 | *C6orf155* | 279 | 4 | 1.01 | Hyper | cg00920327 | 2.74E-03 | 5.24E-07 | 7.06E-04 |
| 7 | Chr7:129007902-129008408 | *AHCYL2* | 506 | 4 | 0.38 | Hypo | cg17827670 | 1.15E-04 | 9.32E-06 | 6.91E-03 |
| 7 | Chr7:1961785-1961870 | *MAD1L1* | 85 | 2 | 0.84 | Hyper | cg15997393 | 2.64E-04 | 3.40E-06 | 1.49E-02 |
| 8 | Chr8:145018010-145018301 | *PLEC1* | 291 | 5 | 0.11 | Hypo | cg20154947 | 4.34E-06 | 1.13E-07 | 1.46E-04 |
| 8 | Chr8:41583321-41583524 | *ANK1* | 203 | 3 | 1.25 | Hyper | cg19537719 | 6.04E-04 | 2.74E-05 | 4.95E-02 |
| 8 | Chr8:48675647-48676055 | *Intergenic* | 408 | 6 | 1.06 | Hyper | cg24160421 | 1.85E-03 | 2.94E-05 | 2.68E-02 |
| 10 | Chr10:121356513-121356866 | *TIAL1* | 353 | 9 | 0.11 | Hypo | cg15856091 | 5.15E-04 | 8.38E-06 | 8.90E-03 |
| 10 | Chr10:123734658-123734890 | *NSMCE4A* | 232 | 4 | 0.06 | Hypo | cg07011445 | 3.85E-03 | 2.75E-05 | 4.36E-02 |
| 10 | Chr10:6214016-6214080 | *PFKFB3* | 64 | 3 | 0.92 | Hypo | cg26262157 | 8.22E-05 | 2.68E-08 | 1.58E-04 |
| 10 | Chr10:74057705-74058093 | *Intergenic* | 388 | 5 | 0.35 | Hypo | cg02190572 | 1.70E-03 | 6.37E-06 | 6.16E-03 |
| 11 | Chr11:1029029-1029337 | *MUC6* | 308 | 5 | 0.19 | Hypo | cg20736321 | 2.65E-05 | 8.50E-06 | 1.03E-02 |
| 11 | Chr11:1036471-1036866 | *MUC6* | 395 | 8 | 0.70 | Hyper | cg22685816 | 5.32E-03 | 4.35E-05 | 4.06E-02 |
| 11 | Chr11:124294778-124295016 | *OR8B4* | 238 | 2 | 0.35 | Hypo | cg24653728 | 4.08E-04 | 2.59E-05 | 4.02E-02 |
| 11 | Chr11:1769289-1769523 | *HCCA2* | 234 | 7 | 0.98 | Hyper | cg03300078 | 6.93E-04 | 3.27E-06 | 5.25E-03 |
| 11 | Chr11:1778524-1778628 | *HCCA2* | 104 | 3 | 0.77 | Hyper | cg22680424 | 2.16E-06 | 7.62E-07 | 2.75E-03 |
| 11 | Chr11:68607622-68608226 | *CPT1A* | 604 | 4 | 0.49 | Hypo | cg00574958 | 1.20E-08 | 1.79E-12 | 1.11E-09 |
| 12 | Chr12:14926744-14926987 | *H2AFJ* | 243 | 3 | 1.33 | Hyper | cg09163005 | 6.58E-03 | 3.02E-05 | 4.57E-02 |

**Table S5.** (Continued)

| Chr | DMR | Nearest  Gene | Size (bp) | CpG count | % Meth | Direction | Index CpG | Lowest P | P-region | Sidak |
| --- | --- | --- | --- | --- | --- | --- | --- | --- | --- | --- |
| 12 | Chr12:49463725-49464042 | *RHEBL1* | 317 | 9 | 0.09 | Hypo | cg05391628 | 1.89E-03 | 8.75E-06 | 1.03E-02 |
| 12 | Chr12:6642229-6642355 | *GAPDH* | 126 | 2 | 0.50 | Hypo | cg02519286 | 4.63E-05 | 5.64E-07 | 1.68E-03 |
| 12 | Chr12:6881997-6882084 | *LAG3* | 87 | 2 | 0.63 | Hypo | cg01820374 | 1.63E-05 | 1.15E-06 | 4.95E-03 |
| 16 | Chr16:2203008-2203177 | *RAB26* | 169 | 2 | 0.54 | Hypo | cg08958747 | 4.68E-04 | 7.32E-06 | 1.62E-02 |
| 16 | Chr16:3114948-3115287 | *IL32* | 339 | 6 | 0.48 | Hypo | cg26724967 | 6.72E-05 | 7.20E-08 | 7.99E-05 |
| 16 | Chr16:50321678-50322157 | *ADCY7* | 479 | 5 | 0.67 | Hyper | cg06897661 | 4.41E-04 | 6.38E-10 | 5.01E-07 |
| 16 | Chr16:75150456-75150881 | *LDHD* | 425 | 7 | 1.05 | Hypo | cg03991512 | 4.62E-04 | 5.91E-08 | 5.24E-05 |
| 16 | Chr16:87734816-87735078 | *LOC100129637* | 262 | 3 | 0.59 | Hyper | cg04324917 | 2.59E-05 | 4.71E-07 | 6.76E-04 |
| 16 | Chr16:89044523-89044883 | *CBFA2T3* | 360 | 5 | 0.39 | Hypo | cg27292079 | 3.33E-04 | 9.14E-07 | 9.55E-04 |
| 17 | Chr17:26662301-26662585 | *TNFAIP1* | 284 | 7 | 0.07 | Hypo | cg06312003 | 3.03E-03 | 2.88E-05 | 3.75E-02 |
| 17 | Chr17:27052676-27052829 | *TLCD1* | 153 | 2 | 0.96 | Hypo | cg09479241 | 1.57E-04 | 2.69E-06 | 6.60E-03 |
| 17 | Chr17:7832680-7833164 | *KCNAB3* | 484 | 8 | 0.67 | Hypo | cg16513459 | 2.12E-03 | 1.42E-05 | 1.09E-02 |
| 19 | Chr19:13951481-13951482 | *Intergenic* | 1 | 1 | 1.10 | Hypo | cg24704287 | 2.34E-08 | 2.34E-08 | 8.75E-03 |
| 19 | Chr19:38806746-38806875 | *EIF4EBP3* | 129 | 5 | 0.08 | Hypo | cg06476903 | 2.88E-03 | 5.45E-07 | 1.59E-03 |
| 19 | Chr19:39389915-39390199 | *SIRT2* | 284 | 2 | 0.10 | Hypo | cg11396509 | 1.67E-04 | 1.71E-05 | 2.25E-02 |
| 19 | Chr19:47287778-47288264 | *SLC1A5* | 486 | 6 | 0.52 | Hypo | cg22304262 | 1.27E-05 | 1.88E-09 | 1.46E-06 |
| 19 | Chr19:49993865-49994150 | *SNORD34* | 285 | 4 | 0.53 | Hypo | cg08570691 | 2.78E-06 | 6.91E-08 | 9.12E-05 |
| 19 | Chr19:55549590-55549843 | *GP6* | 253 | 6 | 1.06 | Hypo | cg18355337 | 2.40E-03 | 5.45E-06 | 8.07E-03 |
| 19 | Chr19:58790125-58790440 | *ZNF8* | 315 | 9 | 0.12 | Hypo | cg20622311 | 1.48E-03 | 5.93E-06 | 7.05E-03 |
| 20 | Chr20:32700182-32700555 | *EIF2S2* | 373 | 8 | 0.13 | Hypo | cg17055717 | 4.05E-03 | 1.84E-05 | 1.84E-02 |
| 20 | Chr20:47897124-47897452 | *C20orf199* | 328 | 4 | 0.51 | Hypo | cg10177030 | 7.20E-04 | 3.39E-05 | 3.82E-02 |
| 21 | Chr21:35320596-35320668 | *Intergenic* | 72 | 2 | 1.42 | Hypo | cg27037013 | 2.90E-06 | 8.16E-10 | 4.26E-06 |
| 21 | Chr21:35831871-35832165 | *KCNE1* | 294 | 8 | 1.22 | Hypo | cg23908228 | 3.62E-03 | 2.56E-06 | 3.27E-03 |
| 21 | Chr21:43656587-43656588 | *ABCG1* | 1 | 1 | 1.28 | Hyper | cg06500161 | 2.34E-11 | 2.34E-11 | 8.81E-06 |
| 22 | Chr22:22987059-22987127 | *POM121L1P* | 68 | 5 | 0.97 | Hyper | cg25432807 | 9.31E-04 | 1.83E-07 | 1.01E-03 |
| 22 | Chr22:32598479-32598717 | *RFPL2* | 238 | 3 | 0.61 | Hypo | cg03477302 | 2.29E-03 | 2.33E-05 | 3.61E-02 |

## Table S6. Association between DNAm at six CpG sites identified in the meta-EWAS, and clinical phenotypes relevant to T2D among diabetes-free participants in ALSPAC (N=1,002).

DNAm at the individual CpGs was modeled as the continuous exposure, and the trait as the outcome in multivariable linear/logistic regressions adjusted for age and sex. Highlighted in bold are associations with corrected p-value <2.0x10^-3^ or α=0.05/23 independent tests (i.e. total number of traits analyzed).

|  | *TXNIP* (cg19693031) | | | *HDAC4* (cg00144180) | | | *CPT1A* (cg00574958) | | | |
| --- | --- | --- | --- | --- | --- | --- | --- | --- | --- | --- |
|  | Effect† | SE | P | Effect | SE | P | Effect | SE | P |  |
| Age [years]¶ | -0.432 | 0.262 | 9.99E-02 | 1.158 | 0.246 | **3.01E-06** | 0.200 | 0.882 | 8.20E-01 |  |
| BMI [kg/m^2^] | -0.268 | 0.252 | 2.87E-01 | 0.332 | 0.242 | 1.70E-01 | -2.295 | 0.845 | 6.72E-03 |  |
| Waist-circumference [cm] | -0.788 | 0.642 | 2.20E-01 | 0.894 | 0.613 | 1.45E-01 | -7.250 | 2.135 | **7.11E-04** |  |
| Fasting Glucose [mmol/L]* | -0.014 | 0.005 | 2.88E-03 | 0.004 | 0.004 | 3.63E-01 | -0.014 | 0.016 | 3.57E-01 |  |
| 2-hours Glucose [mmol/L]* | -0.014 | 0.005 | 6.74E-03 | 0.003 | 0.005 | 5.20E-01 | -0.026 | 0.017 | 1.35E-01 |  |
| C-reactive Protein [mg/L]* | -0.064 | 0.060 | 2.82E-01 | 0.232 | 0.057 | **5.00E-05** | -0.471 | 0.199 | 1.80E-02 |  |
| Fasting Insulin [µIU/mL]*^a^ | -0.037 | 0.045 | 4.12E-01 | 0.068 | 0.039 | 8.20E-02 | -0.374 | 0.131 | 4.35E-03 |  |
| HOMA-IR*^a^ | -0.047 | 0.048 | 3.33E-01 | 0.074 | 0.042 | 7.84E-02 | -0.383 | 0.139 | 6.01E-03 |  |
| HOMA-B*^a^ | -0.007 | 0.042 | 8.74E-01 | 0.050 | 0.036 | 1.71E-01 | -0.360 | 0.120 | 2.85E-03 |  |
| Cholesterol [mmol/L] | -0.032 | 0.050 | 5.22E-01 | -0.095 | 0.048 | 4.58E-02 | -0.234 | 0.167 | 1.62E-01 |  |
| Triglycerides [mmol/L] * | -0.060 | 0.025 | 1.46E-02 | 0.026 | 0.024 | 2.65E-01 | -0.269 | 0.082 | **1.11E-03** |  |
| HDL [mmol/L] | 0.013 | 0.019 | 5.04E-01 | -0.051 | 0.018 | 5.33E-03 | 0.041 | 0.064 | 5.16E-01 |  |
| LDL [mmol/L] | -0.014 | 0.045 | 7.54E-01 | -0.047 | 0.043 | 2.74E-01 | -0.182 | 0.152 | 2.30E-01 |  |
| SBP [mmHg] | -0.883 | 0.683 | 1.96E-01 | 0.895 | 0.654 | 1.71E-01 | -4.403 | 2.284 | 5.42E-02 |  |
| DBP [mmHg]* | -0.005 | 0.007 | 4.47E-01 | 0.016 | 0.007 | 2.04E-02 | -0.027 | 0.024 | 2.52E-01 |  |
| CD8⁺ T cells (%) | 0.001 | 0.002 | 4.65E-01 | 0.003 | 0.002 | 4.02E-02 | 0.000 | 0.005 | 9.34E-01 |  |
| **CD4⁺ T cells (%)** | -0.009 | 0.003 | **1.85E-03** | -0.047 | 0.002 | **9.99E-71** | 0.032 | 0.010 | **1.51E-03** |  |
| Natural Killer Cells (%) | -0.002 | 0.003 | 4.63E-01 | -0.019 | 0.003 | **1.31E-11** | 0.033 | 0.010 | **7.83E-04** |  |
| B cells (%) | 0.001 | 0.002 | 7.50E-01 | -0.018 | 0.001 | **3.99E-32** | 0.020 | 0.005 | **1.65E-04** |  |
| Monocytes (%) | -0.001 | 0.002 | 4.11E-01 | 0.010 | 0.001 | **1.01E-10** | 0.004 | 0.005 | 4.38E-01 |  |
| Granulocytes (%) | 0.012 | 0.005 | 1.32E-02 | 0.061 | 0.004 | **1.90E-45** | -0.077 | 0.016 | **8.57E-07** |  |
| Categorical Phenotypes |  |  |  |  |  |  |  |  |  |  |
| **Sex¶** | 0.364 | 1.150 | **4.44E-13** | 2.001 | 1.142 | **1.69E-07** | 0.025 | 1.724 | **1.50E-11** |  |
| Complete sample |  |  |  |  |  |  |  |  |  |  |
| **Glucose Tolerance** | Mean DNAm%, (SD) | %Meth‡ | P | Mean DNAm%, (SD) | %Meth‡ | P | Mean DNAm%, (SD) | %Meth‡ | P |  |
| *Controls* | 74 (6) | Ref | Ref | 79 (6) | Ref | Ref | 5 (2) | Ref | Ref |  |
| *Prediabetes* | 72 (6) | -2.10% | 1.17E-06 | 81 (6) | 1.80% | 7.17E-05 | 4 (2) | -0.50% | 4.27E-04 |  |
| *T2D cases* | 70 (7) | -3.30% | 2.34E-03 | 83 (6) | 3.50% | 1.13E-03 | 4 (2) | -0.60% | 1.09E-01 |  |
| ***P for trend*** |  |  | **4.11E-08** |  |  | **9.85E-07** |  |  | **1.89E-04** |  |

**Table S6** (Continued)

|  | *SYNM* (cg16765088) | | | *MIR23A* (cg24704287) | | | *ABCG1* (cg06500161) | | |
| --- | --- | --- | --- | --- | --- | --- | --- | --- | --- |
|  | Effect† | SE | P | Effect | SE | P | Effect | SE | P |
| Age [years]¶ | -1.306 | 0.253 | **3.06E-07** | 0.049 | 0.274 | 8.58E-01 | -0.517 | 0.299 | 8.47E-02 |
| BMI [kg/m^2^] | 0.012 | 0.251 | 9.62E-01 | -0.241 | 0.264 | 3.62E-01 | 1.423 | 0.285 | **6.74E-07** |
| Waist-circumference [cm] | 0.053 | 0.636 | 9.33E-01 | -0.332 | 0.668 | 6.19E-01 | 4.216 | 0.719 | **6.28E-09** |
| Fasting Glucose [mmol/L]* | -0.001 | 0.005 | 8.11E-01 | 0.001 | 0.005 | 7.90E-01 | 0.014 | 0.005 | 6.91E-03 |
| 2-hours Glucose [mmol/L]* | -0.007 | 0.005 | 1.55E-01 | -0.002 | 0.005 | 7.81E-01 | 0.011 | 0.006 | 7.02E-02 |
| C-reactive Protein [mg/L]* | 0.012 | 0.059 | 8.45E-01 | -0.134 | 0.062 | 3.06E-02 | 0.227 | 0.068 | **8.66E-04** |
| Fasting Insulin [µIU/mL]*^a^ | -0.021 | 0.043 | 6.27E-01 | -0.038 | 0.044 | 3.86E-01 | 0.202 | 0.047 | **2.22E-05** |
| HOMA-IR*^a^ | -0.018 | 0.046 | 6.89E-01 | -0.035 | 0.047 | 4.55E-01 | 0.212 | 0.050 | **2.77E-05** |
| HOMA-B*^a^ | -0.029 | 0.040 | 4.69E-01 | -0.053 | 0.040 | 1.87E-01 | 0.168 | 0.044 | **1.28E-04** |
| Cholesterol [mmol/L] | -0.075 | 0.049 | 1.29E-01 | 0.032 | 0.052 | 5.45E-01 | 0.042 | 0.057 | 4.68E-01 |
| Triglycerides [mmol/L] * | -0.033 | 0.024 | 1.70E-01 | -0.012 | 0.026 | 6.44E-01 | 0.148 | 0.028 | **1.15E-07** |
| HDL [mmol/L] | -0.001 | 0.019 | 9.48E-01 | 0.044 | 0.020 | 2.67E-02 | -0.104 | 0.021 | **1.55E-06** |
| LDL [mmol/L] | -0.037 | 0.045 | 4.15E-01 | 0.013 | 0.047 | 7.75E-01 | 0.058 | 0.052 | 2.59E-01 |
| SBP [mmHg] | 0.816 | 0.681 | 2.31E-01 | -1.515 | 0.711 | 3.35E-02 | 1.003 | 0.779 | 1.99E-01 |
| DBP [mmHg]* | 0.008 | 0.007 | 2.82E-01 | -0.017 | 0.007 | 2.35E-02 | 0.006 | 0.008 | 4.33E-01 |
| CD8⁺ T cells (%) | -0.010 | 0.002 | **4.79E-10** | -0.004 | 0.002 | 7.39E-03 | 0.000 | 0.002 | 9.74E-01 |
| CD4⁺ T cells (%) | -0.007 | 0.003 | 1.56E-02 | 0.050 | 0.003 | **1.96E-64** | 0.019 | 0.003 | **1.67E-08** |
| Natural Killer Cells (%) | -0.015 | 0.003 | **1.04E-07** | 0.019 | 0.003 | **2.17E-10** | 0.009 | 0.003 | **7.62E-03** |
| B cells (%) | -0.002 | 0.002 | 2.33E-01 | 0.018 | 0.002 | **1.61E-27** | 0.004 | 0.002 | 5.02E-02 |
| Monocytes (%) | 0.007 | 0.002 | **1.40E-06** | -0.008 | 0.002 | **9.93E-07** | 0.002 | 0.002 | 2.22E-01 |
| Granulocytes (%) | 0.022 | 0.005 | **1.55E-06** | -0.064 | 0.004 | **4.24E-43** | -0.032 | 0.005 | **3.12E-09** |
| Categorical Phenotypes |  |  |  |  |  |  |  |  |  |
| Sex¶ | 1.726 | 1.145 | **5.85E-05** | 0.990 | 1.154 | 9.42E-01 | 5.178 | 1.194 | **1.60E-20** |
| Complete sample |  |  |  |  |  |  |  |  |  |
| Glucose Tolerance | Mean DNAm%, (SD) | %Meth‡ | P | Mean DNAm%, (SD) | %Met‡ | P | Mean DNAm%, (SD) | %Meth‡ | P |
| *Controls* | 45 (6) | Ref | Ref | 34 (5) | Ref | Ref | 56 (5) | Ref | Ref |
| *Prediabetes* | 45 (6) | -0.04% | 9.96E-01 | 34 (5) | -0.30% | 7.41E-01 | 58 (6) | 1.50% | 9.18E-05 |
| *T2D cases* | 42 (7) | -2.90% | 7.60E-03 | 32 (6) | -1.90% | 1.05E-01 | 61 (5) | 4.40% | 3.75E-07 |
| ***P for trend*** |  |  | 1.06E-02 |  |  | 1.10E-01 |  |  | **1.47E-09** |

* Variables log-transformed before the analysis. ^a^ Variables available in a subset of 622 control females in ALSPAC. ¶ Associations with age were adjusted for sex only; associations with sex were adjusted for age only. † Effect estimate interpreted as a unit change in the trait, per 10% increase in DNAm at the single CpG site. ‡ Relative percent change in DNA methylation β-values between controls (Ref.) and additional categories of glucose tolerance. *P trend* calculated using an adjusted ANOVA. Categories of glucose tolerance defined by the ADA: Prediabetes if fasting glucose ≥ 5.6 mmol/L & < 7.0 mmol/L (n=199); diabetes if fasting glucose ≥ 7.0 mmol/L (n= 48); controls if fasting glucose < 5.6 mmol/L (n=803).

## Table S7. Association between quartiles of DNAm at cg19693031 (*TXNIP*), and different sociodemographic, anthropometric and metabolic factors of relevance in T2D.

Analyses were adjusted for age and sex and restricted to diabetes-free participants in ALSPAC (N=1,002). Continuous variables were described using the mean and standard deviation (SD) per quartile, while categorical variables were described using proportions per category per quartile. Highlighted in bold are associations where significant difference was observed in the distribution of traits between the lower (reference group) and the top quartile of DNAm at p-value <2.0x10^-3^ (α=0.05/23 traits tested). Quartiles were arranged to represent an increase in the levels of DNAm at the CpG site.

|  | Quartile 1 | Quartile 2 | Quartile 3 | Quartile 4 |  |
| --- | --- | --- | --- | --- | --- |
|  | (n=251) | (n=250) | (n=250) | (n=250) |  |
|  | Mean (SD) | Mean (SD) | Mean (SD) | Mean (SD) | P |
| Age [years]¶ | 51.69(5.48) | 49.95(5.45) | 49.08(4.85) | 49.14(4.93) | 1.25E-01 |
| BMI [kg/m^2^] | 26.9(4.32) | 26.37(4.37) | 26.14(4.15) | 26.37(5.09) | 6.86E-01 |
| Waist-circumference [cm] | 91.73(12.91) | 88.59(13.13) | 86.54(11.53) | 86.46(13.54) | 6.96E-01 |
| Fasting Glucose [mmol/L]* | 5.42(0.5) | 5.29(0.44) | 5.21(0.46) | 5.19(0.41) | 4.56E-03 |
| 2-hours Glucose [mmol/L]* | 4.28(0.41) | 4.26(0.36) | 4.26(0.41) | 4.23(0.39) | 2.67E-03 |
| C-reactive Protein [mg/L]* | 1.91(2.43) | 2.1(2.96) | 2.06(3.27) | 1.73(2.39) | 4.26E-01 |
| Fasting Insulin [µIU/mL]*^a^ | 5.39(3.59) | 5.54(3.62) | 5.61(4.1) | 5.37(3.92) | 7.65E-01 |
| HOMA-IR*^a^ | 1.28(0.94) | 1.29(0.92) | 1.29(0.94) | 1.26(1.04) | 6.54E-01 |
| HOMA-B*^a^ | 61.92(35.17) | 67.21(40.68) | 75.61(93.65) | 63.63(36.1) | 8.11E-01 |
| Cholesterol [mmol/L] | 5.02(0.93) | 4.76(0.97) | 4.8(0.84) | 4.76(0.9) | 6.91E-01 |
| Triglycerides [mmol/L] * | 1.32(0.76) | 1.16(0.66) | 1.09(0.56) | 1.04(0.5) | 7.40E-02 |
| HDL [mmol/L] | 1.37(0.31) | 1.4(0.34) | 1.43(0.35) | 1.46(0.38) | 6.49E-01 |
| LDL [mmol/L] | 3.15(0.82) | 3(0.82) | 3.06(0.77) | 3.02(0.81) | 9.31E-01 |
| SBP [mmHg] | 126.93(14.38) | 123.01(13) | 121.78(13.64) | 119.54(14) | 7.96E-02 |
| DBP [mmHg]* | 75.12(10.13) | 74.25(9.44) | 74.01(12.27) | 72.44(9.41) | 4.08E-01 |
| CD8⁺ T cells | 0.02(0.03) | 0.02(0.03) | 0.01(0.03) | 0.02(0.03) | 5.33E-01 |
| CD4⁺ T cells | 0.18(0.06) | 0.17(0.06) | 0.17(0.05) | 0.17(0.05) | **4.26E-04** |
| Natural Killer Cells | 0.21(0.06) | 0.2(0.05) | 0.2(0.05) | 0.2(0.05) | 5.31E-01 |
| B cells | 0.1(0.03) | 0.09(0.03) | 0.1(0.03) | 0.1(0.03) | 9.75E-01 |
| Monocytes | 0.08(0.03) | 0.07(0.03) | 0.07(0.03) | 0.07(0.03) | 7.08E-01 |
| Granulocytes | 0.49(0.08) | 0.51(0.09) | 0.52(0.08) | 0.52(0.08) | 9.29E-03 |
| Categorical Phenotypes |  |  |  |  |  |
| Sex [females/males]¶ | 97/154 | 152/98 | 181/69 | 191/59 | **9.04E-11** |
| Complete sample |  |  |  |  |  |
| Glucose Tolerance [controls/prediabetes/diabetes] | 165/80/17 | 191/69/2 | 210/42/9 | 212/44/6 | 2.92E-02 |

^a^ Variables measured in a subset of 622 control females in ALSPAC. * Variables log-transformed before the analysis. Categories of diabetes risk defined by the ADA: Prediabetes if fasting glucose ≥ 5.6 mmol/L & < 7.0 mmol/L (n=199); diabetes if fasting glucose ≥ 7.0 mmol/L (n= 48); controls if fasting glucose < 5.6 mmol/L (n=803). ¶ Associations with age were adjusted for sex only; associations with sex were adjusted for age only

## **Table** S8. Association between quartiles of DNAm at cg00144180 (*HDAC4*), and different sociodemographic, anthropometric and metabolic factors of relevance in T2D.

Analyses were adjusted for age and sex and restricted to diabetes-free participants in ALSPAC (N=1,002). Highlighted in bold are associations where significant difference was observed in the distribution of traits between the lower (reference group) and the top quartile of DNAm at p-value <2.0x10^-3^ (α=0.05/23 traits tested).

|  | Quartile 1 | Quartile 2 | Quartile 3 | Quartile 4 |  |
| --- | --- | --- | --- | --- | --- |
|  | (n=251) | (n=250) | (n=250) | (n=251) |  |
|  | Mean (SD) | Mean (SD) | Mean (SD) | Mean (SD) | P |
| Age [years]¶ | 48.41(4.65) | 49.44(4.34) | 50.04(5.51) | 51.97(5.86) | **1.85E-06** |
| BMI [kg/m^2^] | 26.03(4.74) | 26.33(4.43) | 26.93(4.87) | 26.46(3.9) | 5.71E-01 |
| Waist-circumference [cm] | 85.45(13.54) | 87.7(12.62) | 89.47(13.3) | 90.62(11.81) | 3.44E-01 |
| Fasting Glucose [mmol/L]* | 5.21(0.41) | 5.25(0.45) | 5.28(0.48) | 5.35(0.5) | 6.70E-01 |
| 2-hours Glucose [mmol/L]* | 4.27(0.39) | 4.25(0.38) | 4.26(0.4) | 4.24(0.4) | 9.15E-01 |
| C-reactive Protein [mg/L]* | 1.82(3.12) | 1.89(2.61) | 2.13(3.19) | 1.98(2.12) | **1.03E-03** |
| Fasting Insulin [µIU/mL]*^a^ | 5.28(4) | 5.52(3.99) | 5.58(3.52) | 5.64(3.78) | 1.59E-01 |
| HOMA-IR*^a^ | 1.23(1.01) | 1.28(0.94) | 1.31(0.9) | 1.33(0.99) | 1.79E-01 |
| HOMA-B*^a^ | 68.85(84.14) | 68.31(53.57) | 65.94(37.44) | 67.19(37.83) | 1.51E-01 |
| Cholesterol [mmol/L] | 4.81(0.92) | 4.81(0.93) | 4.8(0.89) | 4.91(0.93) | 9.00E-02 |
| Triglycerides [mmol/L] * | 1.1(0.66) | 1.09(0.54) | 1.17(0.62) | 1.25(0.69) | 5.20E-01 |
| HDL [mmol/L] | 1.48(0.37) | 1.44(0.36) | 1.39(0.35) | 1.35(0.3) | 4.15E-03 |
| LDL [mmol/L] | 3.05(0.86) | 3.04(0.85) | 3.04(0.77) | 3.11(0.75) | 4.03E-01 |
| SBP [mmHg] | 120.13(12.65) | 121.77(13.73) | 122.11(13.51) | 127.17(15.07) | 1.63E-01 |
| DBP [mmHg]* | 72.49(10.59) | 73.84(10.04) | 73.28(9.74) | 76.17(10.91) | 3.60E-02 |
| CD8⁺ T cells | 0.01(0.03) | 0.01(0.03) | 0.02(0.03) | 0.02(0.03) | 5.49E-02 |
| CD4⁺ T cells | 0.2(0.05) | 0.18(0.05) | 0.16(0.04) | 0.13(0.05) | **5.99E-58** |
| Natural Killer Cells | 0.21(0.05) | 0.2(0.05) | 0.2(0.05) | 0.19(0.06) | **7.89E-09** |
| B cells | 0.11(0.03) | 0.1(0.03) | 0.09(0.03) | 0.08(0.03) | **2.73E-29** |
| Monocytes | 0.06(0.03) | 0.07(0.03) | 0.07(0.03) | 0.08(0.03) | **2.19E-08** |
| Granulocytes | 0.47(0.08) | 0.5(0.08) | 0.52(0.07) | 0.54(0.09) | **5.72E-37** |
| Categorical Phenotypes |  |  |  |  |  |
| Sex [females/males]¶ | 193/58 | 169/81 | 150/100 | 110/141 | **2.99E-06** |
| Complete sample |  |  |  |  |  |
| Glucose Tolerance [controls/prediabetes/ diabetes] | 213/47/3 | 203/52/7 | 190/60/12 | 173/76/14 | 1.72E-01 |

^a^ Variables measured in a subset of 622 control females in ALSPAC. * Variables log-transformed before the analysis. Categories of diabetes risk defined by the ADA: Prediabetes if fasting glucose ≥ 5.6 mmol/L & < 7.0 mmol/L (n=199); diabetes if fasting glucose ≥ 7.0 mmol/L (n= 48); controls if fasting glucose < 5.6 mmol/L (n=803). ¶ Associations with age were adjusted for sex only; associations with sex were adjusted for age only.

## Table S9. Association between quartiles of DNAm at cg00574958 (*CPT1A*), and different sociodemographic, anthropometric and metabolic factors of relevance in T2D.

Analyses were adjusted for age and sex and restricted to diabetes-free participants in ALSPAC (N=1,002). Highlighted in bold are associations where significant difference was observed in the distribution of traits between the lower (reference group) and the top quartile of DNAm at p-value <2.0x10^-3^ (α=0.05/23 traits tested).

|  | Quartile 1 | Quartile 2 | Quartile 3 | Quartile 4 |  |
| --- | --- | --- | --- | --- | --- |
|  | (n=248) | (n=247) | (n=247) | (n=247) |  |
|  | Mean (SD) | Mean (SD) | Mean (SD) | Mean (SD) | P |
| Age [years]¶ | 51.37(5.47) | 49.71(5.26) | 49.64(5.26) | 49.09(4.94) | 3.12E-01 |
| BMI [kg/m^2^] | 27.26(4.74) | 26.74(4.35) | 26.36(4.4) | 25.5(4.38) | **3.17E-04** |
| Waist-circumference [cm] | 92.51(13.03) | 89.42(12.42) | 88.11(12.83) | 83.52(11.95) | **2.78E-05** |
| Fasting Glucose [mmol/L]* | 5.38(0.5) | 5.28(0.5) | 5.24(0.45) | 5.19(0.39) | 8.31E-02 |
| 2-hours Glucose [mmol/L]* | 4.27(0.39) | 4.26(0.39) | 4.24(0.4) | 4.25(0.39) | **3.33E-02** |
| C-reactive Protein [mg/L]* | 2.2(2.95) | 2.17(3.16) | 1.95(2.7) | 1.49(2.19) | **1.70E-04** |
| Fasting Insulin [µIU/mL]*^a^ | 6.64(4) | 5.51(3.82) | 5.25(4.21) | 5.01(3.31) | **7.92E-06** |
| HOMA-IR*^a^ | 1.55(0.94) | 1.3(1.04) | 1.22(1.01) | 1.17(0.83) | **1.24E-05** |
| HOMA-B*^a^ | 82.82(78.15) | 66.36(36.64) | 68.76(83.05) | 59.95(34.3) | **1.57E-05** |
| Cholesterol [mmol/L] | 5.02(0.93) | 4.89(0.94) | 4.8(0.89) | 4.65(0.87) | 8.95E-02 |
| Triglycerides [mmol/L] * | 1.35(0.65) | 1.21(0.69) | 1.09(0.65) | 0.97(0.45) | **6.41E-07** |
| HDL [mmol/L] | 1.33(0.32) | 1.41(0.35) | 1.46(0.34) | 1.47(0.37) | 5.58E-03 |
| LDL [mmol/L] | 3.18(0.81) | 3.1(0.82) | 3.06(0.79) | 2.92(0.79) | 7.16E-02 |
| SBP [mmHg] | 126.38(13.77) | 123.33(12.86) | 123.14(15.27) | 118.47(12.95) | 2.12E-02 |
| DBP [mmHg]* | 74.9(9.61) | 74.68(10.58) | 74.3(11.27) | 71.82(9.56) | 9.60E-02 |
| CD8⁺ T cells | 0.02(0.03) | 0.01(0.03) | 0.02(0.03) | 0.01(0.03) | 5.18E-01 |
| CD4⁺ T cells | 0.16(0.05) | 0.17(0.05) | 0.17(0.05) | 0.18(0.05) | **3.18E-05** |
| Natural Killer Cells | 0.19(0.06) | 0.2(0.05) | 0.2(0.05) | 0.21(0.05) | **1.42E-05** |
| B cells | 0.09(0.03) | 0.1(0.03) | 0.1(0.03) | 0.1(0.03) | **1.73E-05** |
| Monocytes | 0.08(0.03) | 0.07(0.03) | 0.07(0.03) | 0.07(0.03) | 4.39E-01 |
| Granulocytes | 0.52(0.09) | 0.51(0.08) | 0.5(0.08) | 0.5(0.08) | **6.98E-08** |
| Categorical Phenotypes |  |  |  |  |  |
| Sex [females/males]¶ | 105/143 | 150/97 | 160/87 | 195/52 | **6.74E-11** |
| Complete sample |  |  |  |  |  |
| Glucose Tolerance [controls/prediabetes/ diabetes] | 175/73/11 | 184/65/10 | 191/58/10 | 220/35/4 | 1.78E-02 |

^a^ Variables measured in a subset of 622 control females in ALSPAC. * Variables log-transformed before the analysis. Categories of diabetes risk defined by the ADA: Prediabetes if fasting glucose ≥ 5.6 mmol/L & < 7.0 mmol/L (n=199); diabetes if fasting glucose ≥ 7.0 mmol/L (n= 48); controls if fasting glucose < 5.6 mmol/L (n=803). ¶ Associations with age were adjusted for sex only; associations with sex were adjusted for age only.

## Table S10. Association between quartiles of DNAm at cg16765088 (near *SYNM*), and different sociodemographic, anthropometric and metabolic factors of relevance in T2D.

Analyses were adjusted for age and sex and restricted to diabetes-free participants in ALSPAC (N=1,002). Highlighted in bold are associations where significant difference was observed in the distribution of traits between the lower (reference group) and the top quartile of DNAm at p-value <2.0x10^-3^ (α=0.05/23 traits tested).

|  | Quartile 1 | Quartile 2 | Quartile 3 | Quartile 4 |  |
| --- | --- | --- | --- | --- | --- |
|  | (n=250) | (n=250) | (n=250) | (n=250) |  |
|  | Mean (SD) | Mean (SD) | Mean (SD) | Mean (SD) | P |
| Age [years]¶ | 50.91(5.57) | 49.85(4.94) | 49.57(5) | 49.46(5.46) | **5.93E-06** |
| BMI [kg/m^2^] | 26.4(4.34) | 26.19(4.22) | 26.77(4.7) | 26.39(4.76) | 4.85E-01 |
| Waist-circumference [cm] | 88.19(12.83) | 87.46(12.28) | 88.59(13.37) | 89(13.41) | 6.42E-01 |
| Fasting Glucose [mmol/L]* | 5.28(0.49) | 5.26(0.44) | 5.29(0.46) | 5.28(0.46) | 9.03E-01 |
| 2-hours Glucose [mmol/L]* | 4.28(0.4) | 4.26(0.38) | 4.25(0.39) | 4.23(0.4) | 3.02E-01 |
| C-reactive Protein [mg/L]* | 2.11(3.23) | 1.98(3.04) | 1.87(2.44) | 1.87(2.36) | 5.83E-01 |
| Fasting Insulin [µIU/mL]*^a^ | 5.86(4.73) | 4.95(2.54) | 5.55(4.15) | 5.6(3.61) | 4.63E-01 |
| HOMA-IR*^a^ | 1.36(1.11) | 1.15(0.65) | 1.31(1.09) | 1.32(0.93) | 5.20E-01 |
| HOMA-B*^a^ | 73.71(78.22) | 60.35(28.95) | 70.21(75.57) | 66.83(38.15) | 3.08E-01 |
| Cholesterol [mmol/L] | 4.89(0.91) | 4.83(0.93) | 4.83(0.92) | 4.79(0.91) | 2.01E-01 |
| Triglycerides [mmol/L] * | 1.2(0.68) | 1.14(0.68) | 1.11(0.56) | 1.16(0.61) | 2.46E-01 |
| HDL [mmol/L] | 1.41(0.33) | 1.46(0.38) | 1.4(0.34) | 1.39(0.34) | 7.53E-01 |
| LDL [mmol/L] | 3.09(0.77) | 3.02(0.84) | 3.09(0.83) | 3.04(0.78) | 5.43E-01 |
| SBP [mmHg] | 121.52(13.09) | 122.36(14.27) | 124.13(14.67) | 123.21(13.93) | 3.38E-01 |
| DBP [mmHg]* | 73.11(8.57) | 73.4(10.01) | 74.72(10.4) | 74.58(12.32) | 4.75E-01 |
| CD8⁺ T cells | 0.02(0.04) | 0.02(0.03) | 0.02(0.02) | 0.01(0.02) | **1.08E-08** |
| CD4⁺ T cells | 0.18(0.06) | 0.17(0.05) | 0.17(0.06) | 0.16(0.05) | **2.06E-02** |
| Natural Killer Cells | 0.22(0.06) | 0.2(0.05) | 0.19(0.05) | 0.19(0.05) | **2.03E-09** |
| B cells | 0.1(0.03) | 0.1(0.03) | 0.09(0.03) | 0.09(0.03) | 1.25E-01 |
| Monocytes | 0.07(0.03) | 0.07(0.03) | 0.07(0.03) | 0.08(0.03) | **1.55E-07** |
| Granulocytes | 0.49(0.09) | 0.51(0.09) | 0.51(0.08) | 0.52(0.07) | **1.28E-06** |
| Categorical Phenotypes |  |  |  |  |  |
| Sex [females/males]¶ | 160/90 | 165/85 | 156/94 | 140/110 | **1.47E-04** |
| Complete sample |  |  |  |  |  |
| Glucose Tolerance [controls/prediabetes/ diabetes] | 187/63/12 | 200/52/10 | 197/58/7 | 194/61/7 | 4.02E-01 |

^a^ Variables measured in a subset of 622 control females in ALSPAC. * Variables log-transformed before the analysis. Categories of diabetes risk defined by the ADA: Prediabetes if fasting glucose ≥ 5.6 mmol/L & < 7.0 mmol/L (n=199); diabetes if fasting glucose ≥ 7.0 mmol/L (n= 48); controls if fasting glucose < 5.6 mmol/L (n=803). ¶ Associations with age were adjusted for sex only; associations with sex were adjusted for age only.

## Table S11. Association between quartiles of DNAm at cg24704287 (near *MIR23A*), and different sociodemographic, anthropometric and metabolic factors of relevance in T2D.

Analyses were adjusted for age and sex and restricted to diabetes-free participants in ALSPAC (N=1,002). Highlighted in bold are associations where significant difference was observed in the distribution of traits between the lower (reference group) and the top quartile of DNAm at p-value <2.0x10^-3^ (α=0.05/23 traits tested).

|  | Quartile 1 | Quartile 2 | Quartile 3 | Quartile 4 |  |
| --- | --- | --- | --- | --- | --- |
|  | (n=251) | (n=250) | (n=250) | (n=251) |  |
|  | Mean (SD) | Mean (SD) | Mean (SD) | Mean (SD) | P |
| Age [years]¶ | 49.96(5.43) | 50.05(5.71) | 49.81(4.81) | 50.05(5.18) | 7.04E-01 |
| BMI [kg/m^2^] | 27.01(4.95) | 25.98(4.07) | 26.17(4.21) | 26.59(4.69) | 3.05E-01 |
| Waist-circumference [cm] | 89.49(13.56) | 87.34(11.97) | 87.88(12.9) | 88.52(13.31) | 5.13E-01 |
| Fasting Glucose [mmol/L]* | 5.28(0.5) | 5.28(0.46) | 5.23(0.43) | 5.31(0.47) | 2.81E-01 |
| 2-hours Glucose [mmol/L]* | 4.26(0.4) | 4.27(0.41) | 4.21(0.38) | 4.28(0.39) | 6.32E-01 |
| C-reactive Protein [mg/L]* | 2.29(3.1) | 1.6(2.05) | 1.91(2.99) | 2.01(2.86) | 3.21E-02 |
| Fasting Insulin [µIU/mL]*^a^ | 5.9(3.97) | 5.2(3.31) | 5.02(3.37) | 5.84(4.56) | 5.76E-01 |
| HOMA-IR*^a^ | 1.37(1.01) | 1.24(0.89) | 1.16(0.84) | 1.36(1.08) | 6.91E-01 |
| HOMA-B*^a^ | 76.67(77.91) | 59.9(30) | 62.17(37.18) | 72.88(77.42) | 2.43E-01 |
| Cholesterol [mmol/L] | 4.84(0.94) | 4.76(0.93) | 4.84(0.91) | 4.89(0.89) | 3.85E-01 |
| Triglycerides [mmol/L] * | 1.21(0.71) | 1.09(0.55) | 1.11(0.49) | 1.21(0.73) | 8.92E-01 |
| HDL [mmol/L] | 1.38(0.33) | 1.43(0.35) | 1.42(0.35) | 1.43(0.36) | 1.54E-01 |
| LDL [mmol/L] | 3.05(0.8) | 3.02(0.82) | 3.08(0.77) | 3.08(0.84) | 5.67E-01 |
| SBP [mmHg] | 125.42(15.22) | 121.12(13.92) | 121.78(12.69) | 122.91(13.76) | 5.79E-02 |
| DBP [mmHg]* | 75.64(10.95) | 72.99(9.84) | 73.3(9.02) | 73.86(11.51) | 7.01E-02 |
| CD8⁺ T cells | 0.02(0.03) | 0.02(0.03) | 0.02(0.03) | 0.01(0.02) | **2.57E-05** |
| CD4⁺ T cells | 0.14(0.05) | 0.16(0.05) | 0.18(0.05) | 0.2(0.05) | **3.29E-52** |
| Natural Killer Cells | 0.18(0.06) | 0.2(0.05) | 0.2(0.05) | 0.21(0.05) | **3.20E-09** |
| B cells | 0.08(0.03) | 0.09(0.02) | 0.1(0.03) | 0.11(0.03) | **4.19E-23** |
| Monocytes | 0.08(0.03) | 0.07(0.03) | 0.07(0.03) | 0.07(0.03) | **2.21E-05** |
| Granulocytes | 0.55(0.09) | 0.52(0.07) | 0.5(0.08) | 0.47(0.07) | **2.63E-33** |
| Categorical Phenotypes |  |  |  |  |  |
| Sex [females/males]¶ | 150/101 | 164/86 | 152/98 | 156/95 | 6.81E-01 |
| Complete sample |  |  |  |  |  |
| Glucose Tolerance [controls/prediabetes/ diabetes] | 189/63/11 | 196/57/9 | 201/49/12 | 193/66/3 | 7.90E-01 |

^a^ Variables measured in a subset of 622 control females in ALSPAC. * Variables log-transformed before the analysis. Categories of diabetes risk defined by the ADA: Prediabetes if fasting glucose ≥ 5.6 mmol/L & < 7.0 mmol/L (n=199); diabetes if fasting glucose ≥ 7.0 mmol/L (n= 48); controls if fasting glucose < 5.6 mmol/L (n=803). ¶ Associations with age were adjusted for sex only; associations with sex were adjusted for age only.

## Table S12. Association between quartiles of DNAm at cg06500161 (*ABCG1*), and different sociodemographic, anthropometric and metabolic factors of relevance in T2D.

Analyses were adjusted for age and sex and restricted to diabetes-free participants in ALSPAC (N=1,002). Highlighted in bold are associations where significant difference was observed in the distribution of traits between the lower (reference group.) and the top quartile of DNAm at p-value <2.0x10^-3^ (α=0.05/23 traits tested).

|  | Quartile 1 | Quartile 2 | Quartile 3 | Quartile 4 |  |
| --- | --- | --- | --- | --- | --- |
|  | (n=251) | (n=250) | (n=250) | (n=250) |  |
|  | Mean (SD) | Mean (SD) | Mean (SD) | Mean (SD) | P |
| Age [years]¶ | 48.93(4.71) | 49.96(5.29) | 50.28(5.53) | 50.69(5.45) | 1.66E-01 |
| BMI [kg/m^2^] | 25.3(3.86) | 26.15(4.49) | 26.4(4.32) | 27.93(4.9) | **3.81E-08** |
| Waist-circumference [cm] | 82.88(10.84) | 87.4(12.56) | 88.46(11.33) | 94.53(14.21) | **1.16E-09** |
| Fasting Glucose [mmol/L]* | 5.16(0.42) | 5.27(0.46) | 5.27(0.47) | 5.39(0.48) | 3.94E-03 |
| 2-hours Glucose [mmol/L]* | 4.27(0.4) | 4.26(0.4) | 4.23(0.37) | 4.27(0.4) | 6.34E-02 |
| C-reactive Protein [mg/L]* | 1.59(2.25) | 1.83(2.69) | 1.95(2.53) | 2.46(3.48) | **2.08E-04** |
| Fasting Insulin [µIU/mL]*^a^ | 4.81(3.43) | 5.11(2.95) | 5.74(3.53) | 7.17(5.65) | **7.93E-07** |
| HOMA-IR*^a^ | 1.11(0.84) | 1.2(0.76) | 1.33(0.88) | 1.71(1.41) | **7.09E-07** |
| HOMA-B*^a^ | 61.62(59.68) | 60.72(31.54) | 75.09(75.48) | 81.35(66.06) | **3.20E-05** |
| Cholesterol [mmol/L] | 4.69(0.87) | 4.79(0.92) | 4.91(0.93) | 4.95(0.93) | 9.47E-01 |
| Triglycerides [mmol/L] * | 0.95(0.49) | 1.12(0.56) | 1.21(0.7) | 1.33(0.7) | **1.30E-05** |
| HDL [mmol/L] | 1.53(0.36) | 1.44(0.34) | 1.39(0.34) | 1.31(0.31) | **8.76E-07** |
| LDL [mmol/L] | 2.95(0.77) | 3.05(0.8) | 3.11(0.82) | 3.13(0.83) | 4.34E-01 |
| SBP [mmHg] | 119.05(12.58) | 121.65(14.59) | 122.58(12.85) | 127.9(14.44) | 3.85E-02 |
| DBP [mmHg]* | 72.81(11.05) | 73.27(10.26) | 73.17(8.39) | 76.51(11.3) | 1.01E-01 |
| CD8⁺ T cells | 0.01(0.02) | 0.02(0.03) | 0.02(0.03) | 0.02(0.03) | 7.08E-01 |
| CD4⁺ T cells | 0.16(0.05) | 0.17(0.05) | 0.17(0.05) | 0.18(0.06) | **2.21E-08** |
| Natural Killer Cells | 0.2(0.05) | 0.19(0.05) | 0.2(0.05) | 0.21(0.05) | 1.66E-02 |
| B cells | 0.1(0.03) | 0.09(0.03) | 0.09(0.03) | 0.1(0.03) | 5.04E-02 |
| Monocytes | 0.07(0.03) | 0.07(0.03) | 0.07(0.03) | 0.08(0.03) | 5.31E-01 |
| Granulocytes | 0.53(0.08) | 0.52(0.09) | 0.5(0.09) | 0.48(0.08) | **1.72E-08** |
| Categorical Phenotypes |  |  |  |  |  |
| Sex [females/males]¶ | 206/45 | 169/81 | 152/98 | 95/155 | **7.48E-19** |
| Complete sample |  |  |  |  |  |
| Glucose Tolerance [controls/prediabetes/ diabetes] | 217/45/1 | 196/59/7 | 197/53/12 | 168/78/16 | 3.50E-02 |

^a^ Variables measured in a subset of 622 control females in ALSPAC. * Variables log-transformed before the analysis. Categories of diabetes risk defined by the ADA: Prediabetes if fasting glucose ≥ 5.6 mmol/L & < 7.0 mmol/L (n=199); diabetes if fasting glucose ≥ 7.0 mmol/L (n= 48); controls if fasting glucose < 5.6 mmol/L (n=803). ¶ Associations with age were adjusted for sex only; associations with sex were adjusted for age only.

|  | # Genes | Genes differentially  methylated | P |
| --- | --- | --- | --- |
| ***GO term*** |  |  |  |
| NAD-dependent protein deacetylase activity | 15 | 3 | 4.22E-05 |
| NAD binding | 50 | 4 | 6.05E-05 |
| GAIT complex | 4 | 2 | 1.09E-04 |
| Glycolytic process | 67 | 4 | 3.02E-04 |
| ATP generation from ADP | 68 | 4 | 3.28E-04 |
| Pyruvate biosynthetic process | 71 | 4 | 3.49E-04 |
| Peptidyl-lysine deacetylation | 6 | 2 | 3.49E-04 |
| Nucleolus | 1155 | 13 | 4.11E-04 |
| ADP metabolic process | 75 | 4 | 4.91E-04 |
| Main axon | 65 | 4 | 5.23E-04 |
| Sarcoplasm | 71 | 4 | 5.53E-04 |
| Nucleoside diphosphate phosphorylation | 83 | 4 | 6.14E-04 |
| Nucleotide phosphorylation | 85 | 4 | 6.75E-04 |
| Negative regulation of cellular carbohydrate metabolic process | 36 | 3 | 7.31E-04 |
| Regulation of small molecule metabolic process | 326 | 7 | 7.35E-04 |
| Purine nucleoside diphosphate metabolic process | 83 | 4 | 7.36E-04 |
| Purine ribonucleoside diphosphate metabolic process | 83 | 4 | 7.36E-04 |
| Ribonucleoside diphosphate metabolic process | 85 | 4 | 8.16E-04 |
| Regulation of glycolytic process | 38 | 3 | 9.07E-04 |
| Histone H4 deacetylation | 10 | 2 | 9.48E-04 |
| ***KEGG pathway*** |  |  |  |
| Nicotinate and nicotinamide metabolism | 35 | 2 | 8.26E-03 |
| Oocyte meiosis | 116 | 3 | 1.50E-02 |
| Cysteine and methionine metabolism | 44 | 2 | 1.53E-02 |
| Platelet activation | 118 | 3 | 1.87E-02 |
| NOD-like receptor signaling pathway | 152 | 3 | 1.91E-02 |
| Estrogen signaling pathway | 127 | 3 | 2.25E-02 |
| Apelin signaling pathway | 130 | 3 | 2.60E-02 |
| Biosynthesis of amino acids | 60 | 2 | 2.66E-02 |
| PPAR signaling pathway | 69 | 2 | 2.95E-02 |
| Adipocytokine signaling pathway | 61 | 2 | 3.20E-02 |
| Cortisol synthesis and secretion | 57 | 2 | 3.76E-02 |
| Thyroid hormone synthesis | 65 | 2 | 4.03E-02 |
| Pathogenic Escherichia coli infection | 186 | 3 | 4.78E-02 |
| Salivary secretion | 83 | 2 | 5.37E-02 |
| Gastric acid secretion | 72 | 2 | 5.39E-02 |
| Pancreatic secretion | 92 | 2 | 5.90E-02 |
| Progesterone-mediated oocyte maturation | 88 | 2 | 6.39E-02 |
| Gap junction | 82 | 2 | 6.46E-02 |
| Carbon metabolism | 98 | 2 | 6.62E-02 |
| GnRH signaling pathway | 89 | 2 | 6.65E-02 |

## Table S13. Enrichment analysis for biological processes associated with six CpG sites identified in the meta-analysis, and 77 index CpGs identified within DMRs.

Top 20 GO terms and KEGG pathways detected with the smallest p-value of enrichment (unadjusted). None of the pathways identified surpassed Bonferroni correction at p<0.05/total number of genes in a pathway. # Genes: total number of genes within a pathway/GO term; Genes differentially methylated: subset of genes identified with differential methylation among genes within a same pathway; P: unadjusted p-value.

## Table S14. Comparison of mean values of DNA methylation between blood and five metabolically relevant tissues for T2D at six CpGs identified in the meta-analysis of EWAS.

Tissue-specific mean values of methylation were retrieved from the publicly available GEO dataset GSE48472 based on the study conducted by Slieker *et al.*[31]. DNAm was measured using the HM450 array in 5 peripheral blood samples (mean age= 28 years) and 6 internal tissue samples (mean age= 66 years) [31].

| CpG | Gene | Blood | Pancreas | Muscle | Subcutaneous Fat | Visceral Fat | Liver |
| --- | --- | --- | --- | --- | --- | --- | --- |
| cg00144180 | *HDAC4* | 0.85 | 0.97 | 0.97 | 0.97 | 0.92 | 0.97 |
| cg00574958 | *CPT1A* | 0.12 | 0.26 | 0.09 | 0.10 | 0.18 | 0.10 |
| cg06500161 | *ABCG1* | 0.67 | 0.53 | 0.38 | 0.47 | 0.59 | 0.68 |
| cg16765088 | *SYNM* | 0.50 | 0.38 | 0.56 | 0.43 | 0.40 | 0.30 |
| cg19693031 | *TXNIP* | 0.72 | 0.22 | 0.70 | 0.54 | 0.54 | 0.30 |
| cg24704287 | *MIR23A* | 0.30 | 0.57 | 0.17 | 0.23 | 0.42 | 0.51 |

## Table S15. List of eQTMs identified in BIOS QTL in association with three of our six T2D-associated CpGs from the meta-analysis.

r: correlation between DNAm and gene expression of the specific gene transcript. All associations surpassed FDR correction (FDR < 0.05).

| CpG | Chr | Gene | Transcript | r | P |
| --- | --- | --- | --- | --- | --- |
| cg19693031 | 1 | *TXNIP* | ENSG00000117289 | -0.12 | 7.14E-08 |
| cg00574958 | 11 | *CPT1A* | ENSG00000110090 | -0.17 | 3.05E-20 |
| cg06500161 | 21 | *ABCG1* | ENSG00000160179 | -0.32 | 2.22E-37 |

## Table S16. List of meQTL identified in Genetics of DNAm Consortium (GoDMC) in association with five of our six T2D-associated CpGs from the meta-analysis.

| CpG | SNP | Class | Gene | Effect  estimate | Effect  Allele | Other  Allele | EAF* | P | N |
| --- | --- | --- | --- | --- | --- | --- | --- | --- | --- |
| cg00144180 | rs11693641 | *Cis* | *HDAC4* | -0.15 | A | C | 0.5 | 3.54E-202 | 23,360 |
| cg00144180 | rs1872614 | *Cis* | *HDAC4* | -0.05 | A | T | 0.6 | 2.22E-06 | 16,512 |
| cg06500161 | rs220182 | *Cis* | *ABCG1* | 0.06 | T | C | 0.6 | 3.54E-202 | 24,474 |
| cg16765088 | rs7496161 | *Cis* | *SYNM* | -0.32 | A | G | 0.1 | 4.70E-122 | 25,984 |
| cg19693031 | rs6657798 | *Trans* | *TXNIP* | -0.46 | C | G | 0.8 | 3.54E-202 | 27,212 |
| cg24704287 | rs36116239 | *Cis* | *MIR23A* | -0.13 | T | C | 0.3 | 3.54E-202 | 20,165 |

*Effect allele frequency.

## **Table S17. Overlap between meQTL associated with differentially methylated CpGs in the meta-EWAS, and GWAS SNPs for different glycemic traits.**

Highlighted in bold are meQTL that overlapped with a GWAS SNP and were identified nominally associated with the trait at GWAS p-value <0.05.

| Trait | Overlapped meQTL  & GWAS SNP | CpG | Effect estimate (GWAS) | Effect Allele (GWAS) | Similar  EA* | P-value (GWAS) |
| --- | --- | --- | --- | --- | --- | --- |
| T2D | --- | --- | --- | --- | --- | --- |
| Fasting insulin | rs220182 | cg06500161 (*ABCG1*) | 1.000 | C | No | 0.22 |
| Fasting glucose | --- | --- | --- | --- | --- | --- |
| HbA1c | rs1872614 | cg00144180 (*HDAC4*) | 0.004 | A | Yes | 0.40 |
|  | rs220182 | cg06500161 (*ABCG1*) | -0.003 | T | Yes | 0.48 |
|  | rs6657798 | cg19693031 (*TXNIP*) | -0.004 | C | Yes | 0.40 |
|  | rs7496161 | cg16765088 (*SYNM*) | 0.001 | A | Yes | 0.89 |
| 2-h glucose | rs1872614 | cg00144180 (*HDAC4*) | -0.014 | A | Yes | 0.68 |
|  | rs220182 | cg06500161 (*ABCG1*) | -0.010 | T | Yes | 0.64 |
|  | **rs6657798** | **cg19693031 (*TXNIP*)** | **0.059** | **C** | **Yes** | **0.01** |
|  | rs7496161 | cg16765088 (*SYNM*) | 0.043 | A | Yes | 0.16 |
| HOMA-IR | rs1872614 | cg00144180 (*HDAC4*) | -0.007 | A | Yes | 0.22 |
|  | rs220182 | cg06500161 (*ABCG1*) | -0.005 | T | Yes | 0.28 |
|  | rs6657798 | cg19693031 (*TXNIP*) | 0.003 | C | Yes | 0.50 |
|  | rs7496161 | cg16765088 (*SYNM*) | -0.005 | A | Yes | 0.43 |
| HOMA-B | rs1872614 | cg00144180 (*HDAC4*) | -0.006 | A | Yes | 0.18 |
|  | **rs220182** | **cg06500161 (*ABCG1*)** | **-0.008** | **T** | **Yes** | **0.02** |
|  | rs6657798 | cg19693031 (*TXNIP*) | -0.003 | C | Yes | 0.53 |
|  | rs7496161 | cg16765088 (*SYNM*) | -0.005 | A | Yes | 0.39 |

*EA: effect allele.
